# Simulated Range Expansion Suggests Rapid Change in Biotic Resistance to a Range‐Shifting Competitor

**DOI:** 10.1002/ece3.74096

**Published:** 2026-07-29

**Authors:** Emma Menchions, Amy Angert

**Affiliations:** ^1^ Department of Botany University of British Columbia Vancouver British Columbia Canada; ^2^ Department of Zoology University of British Columbia Vancouver British Columbia Canada

**Keywords:** biotic resistance, competition, duckweed, phenotypic plasticity, range expansion, rapid evolution

## Abstract

Variable climate‐change‐driven range shifts will likely create novel species and population interactions. Most research has focused on how these interactions may impact expansion rates and adaptation of species on the move (range‐shifters). However, slower‐moving resident species could also respond to novel competitors, as seen in biological invasions. Whether resident adaptation can occur at initial, low‐density phases of these range expansions remains unknown. Here, we simulate this scenario by constructing populations of eight duckweed genotypes ( *Lemna minor* = resident) from various localities near and beyond the range edge of a potential range‐shifting competitor, *Spirodela polyrhiza* , and introduce one genotype of *S. polyrhiza* at low density. After 14 weeks, we observed significant but subtle evidence for rapid evolution and phenotypic plasticity in the resident, including selection for resident genotypes with faster growth. These changes led to an increase in the production of dormant *S. polyrhiza* propagules (turions) when reintroduced to *L. minor* populations. However, whether turion production increases or decreases biotic resistance exerted by *L. minor* populations depends on the contribution of turion production to short‐ vs. long‐term population growth in *S. polyrhiza* . Collectively, these results suggest that range‐shifting species, even at low densities, may drive evolution, plasticity, and evolution of biotic resistance in resident competitors, but further study is needed to confirm the direction of change in biotic resistance as well as the existence and generality of these effects in a natural context.

## Graphical abstract

We constructed populations of *Lemna minor* genotypes sourced from localities near and beyond the range edge of a potential range‐shifting competitor, *Spirodela polyrhiza* . Following 14 weeks of exposure to low densities of *S. polyrhiza* , we observed subtle but significant rapid evolution and phenotypic plasticity of functional traits in *L. minor* , as well as a change in biotic resistance exerted by *L. minor* on *S. polyrhiza* . Collectively, this evidence suggests that range‐shifting species, even at low densities, may drive evolution of traits and trait plasticity that affect biotic resistance, which could feed back to affect the success of the range‐shifter; however, further study is required to confirm the strength and generality of such effects in natural systems.

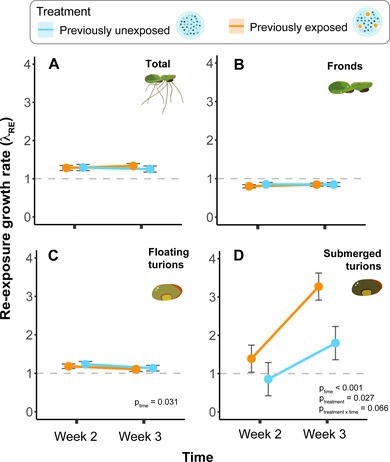

## 1 Introduction

As anthropogenic climate change continues, species ranges are shifting rapidly (Lenoir and Svenning 2015; Parmesan et al. 2022; Urban 2015). Differences in sensitivity to climate change and ability to track shifting suitable climates will likely create novel communities as moving species (range‐shifters) encounter new competitor species or populations (residents) (Alexander et al. 2015; Urban et al. 2012). Most work has focused on predicting range‐shift rates and elucidating eco‐evolutionary dynamics in range‐shifters (Miller et al. 2020; Wallingford et al. 2020). However, resident species may themselves experience evolutionary change as they respond to novel selection pressure from range‐shifting competitors (Zarzyczny et al. 2024). This has been frequently observed in human‐mediated, often intercontinental, biological range expansions, where non‐endemic plant species can drive evolution and phenotypic plasticity affecting the life‐history traits, physiology, allelopathy tolerance, and morphology of resident competitors (Berthon 2015; Case et al. 2016; Strauss et al. 2006). However, these evolutionary impacts are usually documented after non‐endemic plants have reached high abundance within the community, and it remains unknown whether residents can respond during initial, low‐density stages of range expansion. This context is especially relevant to range expansions driven by climate change, where the range‐shifter may frequently disperse beyond its range edge and be less likely to reach a high local abundance due to having some history of parapatry or sympatry with different populations of the resident (Strauss et al. 2006; Zarzyczny et al. 2024).

Following the conceptual framework of the Modern Coexistence Theory, residents may adapt to newly arrived range‐shifters and alter the strength of competitive interactions via two pathways (Aarssen 1983; Chesson 2000): decreased niche overlap (classic character displacement, Schluter (2000)), although convergent and parallel character shifts are plausible (Abrams 1986; Fox and Vasseur 2008; Germain et al. 2018) and/or equalized competitive ability (a cryptic axis of character displacement, Germain et al. (2020)). If residents adapt to newly arrived competitors by decreasing niche overlap, they may become easier to invade (decreased biotic resistance) (Carboni et al. 2013). Alternatively, if competitive ability is initially asymmetric in favor of the range‐shifter, then co‐evolution could yield increased biotic resistance, whereby residents develop greater competitive ability in the presence of the range‐shifter. Therefore, if resident populations can rapidly respond to low densities of a range‐shifting competitor, these changes could feed back to either promote or hinder further range expansion. Such effects have been investigated for more novel competitive interactions between invasive and native grassland species (Ferrero‐Serrano et al. 2011; Germain et al. 2020) and for less novel antagonistic interactions between microbe assemblages (Faillace and Morin 2016), but have not been tested in the context of range expansions under climate change.

Assemblages of macrophytes from the family Lemnaceae (duckweeds) are ideal model systems for investigating such eco‐evolutionary dynamics due to their rapid growth, small size, and propensity for clonal reproduction (Laird and Barks 2018). The latter characteristic allows duckweeds to confer transgenerational plasticity as daughter fronds bud from the parent raft (i.e., cluster of connected fronds that constitute a floating individual ramet) (Hillman 1961; Van Antro et al. 2023). As such, they have been used to study phenotypic plasticity arising from competition (Hess et al. 2022). Additionally, sexual reproduction is rare (Fourounjian et al. 2021), and at least one species has been found to have exceedingly low mutation rates (Sandler et al. 2020; Xu et al. 2019). Combined, these traits make them amenable to studies of rapid evolution (Hart et al. 2019), defined as evolution on timescales relevant to ecological processes (Hairston Jr et al. 2005), as it can be quantified by simply observing changes in genotype frequencies over time (Hart et al. 2019; Laird and Barks 2018).

As natural selection acts on traits, trait differences among genotypes must underlie adaptive evolution driven by competition. Despite the simple morphology of duckweeds, there are still many functional traits that exhibit intraspecific variation for selection to act upon (Smith et al. 2025; Usui and Angert 2024). As with other vascular plants, traits related to resource uptake and allocation (e.g., root depth, frond area, root‐to‐shoot allocation, intrinsic growth rates: Jewell and Bell 2023a; Ziegler et al. 2015) and dispersal (e.g., the timing of daughter frond fragmentation or the number of fronds per duckweed raft: Smith et al. 2025; Zhang et al. 2020) can contribute to net fitness and niche differences between genotypes (Angert et al. 2009; Kraft et al. 2015). By measuring these functional traits in competition experiments, one can, therefore, infer which traits are targeted by competition‐driven selection and which respond to competition through plasticity (Hart et al. 2019).

To investigate the potential for rapid adaptation and altered biotic resistance in a resident following low‐density exposure to a range‐shifting competitor, we conducted proof‐of‐concept competition experiments between duckweeds within the *Lemna minor* L. species complex and *Spirodela polyrhiza* (L.) Schleid. These species naturally compete across most of their broad, overlapping distributions for light, nutrients, and space (Armitage and Jones 2020; Strzałek and Kufel 2021). However, at colder temperatures in high‐latitude regions, such as northern Europe, *L. minor* currently competitively excludes *S. polyrhiza* (Armitage and Jones 2020). With climate change, however, these regions are warming to temperatures that could allow the more southerly *S. polyrhiza* to expand by the end of the century, which would lead to more frequent and longer competitive interactions (Armitage and Jones 2019; European Environment Agency 2026; Climate Impact Lab 2022). This could, in turn, affect the resistance of *L. minor* populations to further expansion by *S. polyrhiza* .

We assembled resident populations of *L. minor* genotypes and experimentally simulated the colonization of *S. polyrhiza* at low density and high temperature that could be experienced near *L. minor* 's range edge to answer the following questions: (Q1) Could low densities of a range‐shifting competitor drive rapid phenotypic change (through genotypic evolution and plasticity) in a resident species? (Q2) Might selection favor particular resident functional traits? (Q3) Could rapid phenotypic change in a resident feed back to affect further expansion success (biotic resistance)?

We first predicted that *L. minor* would rapidly respond to low‐density exposure to *S. polyrhiza*, as rapid evolution (Hart et al. 2019) and phenotypic plasticity (Hess et al. 2022) have been observed following competition between these species within 10–15 generations. We did not form a priori hypotheses regarding our second research question (selection on functional traits), as others have cautioned against using functional traits to predict the outcome of competitive interactions (Levine et al. 2025). Instead, we explored univariate associations between changes in the relative abundance of *L. minor* genotypes and the means and plasticities of five traits. Finally, we predicted that previously exposed *L. minor* populations would support lower growth rates of *S. polyrhiza* upon secondary exposure (increased biotic resistance) because previous studies have shown support for the evolution of increased biotic resistance in biological invasions (Germain et al. 2020; Oduor 2013; Sheppard and Schurr 2019). With this proof‐of‐concept study, we aim to determine whether range‐shifters in initial low‐density expansion phases may drive sufficient, rapid phenotypic changes in resident competitors to affect the movement of the simulated range‐shifter. Determining whether this phenomenon is possible in a simplified setting will establish future research paths toward elucidating the eco‐evolutionary dynamics of competitive interactions during community re‐assembly under climate change.

## 2 Materials and Methods

### 2.1 Genotype Selection

**FIGURE 1:**
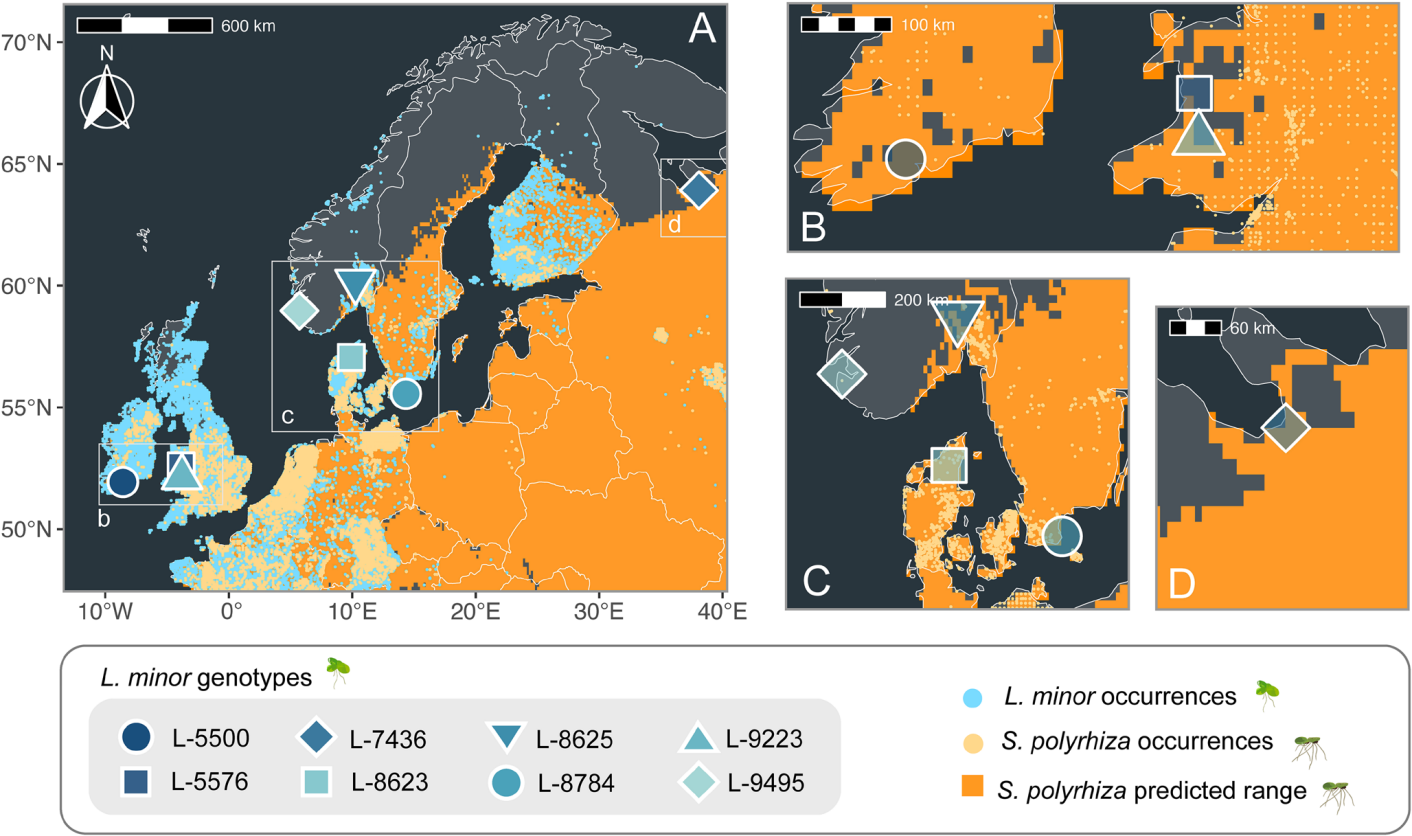
Collection locations of eight genotypes of the duckweed *Lemna minor* selected from the Rutgers Duckweed Stock Cooperative (white outlines, colored shapes) in Northern Europe overlaid on the distribution of known occurrences for *L. minor* (light blue points), *Spirodela polyrhiza* (light orange points), and the predicted range of *S. polyrhiza* (orange) where *S. polyrhiza* is predicted to be able to coexist with *L. minor* (reproduced from Armitage (2020): https://doi.org/10.6084/m9.figshare.12649601.v1). *L. minor* occurrences were removed for clarity in zoomed‐in views (B, C, D). Species occurrences cleaned by Armitage and Jones (2020) were obtained from the Global Biodiversity Information Facility (GBIF) and various botanical societies. Map credits: “rnaturalearth” package.

To assemble resident populations of *L. minor* , we searched the Rutgers Duckweed Stock Cooperative (RDSC) for genotypes that originated from near and beyond the predicted northern range edge of *S. polyrhiza* in Northern Europe (Armitage and Jones 2020) (Figure 1, Table S1). To reduce the chance that an *L. minor* genotype has experienced a consistent recent sympatric history with *S. polyrhiza* , we used occurrences of *S. polyrhiza* , cleaned and provided by Armitage and Jones (2020), to ensure that localities of *L. minor* collections were at least 10 km away from known *S. polyrhiza* occurrences (Table S1). We selected eight genotypes from various localities across Northern Europe identified as *L. minor* in the RDSC stock database that matched this criterion. This diversity lies within the range of genotypic diversity reported in natural populations (between 4 and 20 distinct genotypes per pond) (Bog et al. 2022; Cole and Voskuil 1996; Vasseur et al. 1993; but see Schmid et al. 2024), though drawing from disparate source populations across a large spatial extent is not entirely representative of natural *L. minor* populations (Schmid et al. 2024). Additionally, we selected a range‐shifter strain ( *S. polyrhiza* ) collected from Vancouver, BC, which has a recent sympatric history with *L. minor* genotypes from the region, but likely not with the *L. minor* genotypes from Europe used here.

Our selection of *L. minor* genotypes was complicated by new genetic methods revealing cryptic diversity within accessions previously identified as *L. minor* based on morphology and plastid markers (Braglia, Breviario, et al. 2021; Senevirathna et al. 2021; Michael et al. 2025). In particular, three of the putative *L. minor* accessions we originally selected were recently re‐identified as *Lemna* × *japonica* genotypes, a hybrid between *L. minor* and *L. turionifera* (Braglia, Breviario, et al. 2021; Braglia, Lauria, et al. 2021). Therefore, our constructed resident populations comprised a species complex, including both hybrids (*n* = 3), non‐hybrids (*n* = 4), and an unconfirmed accession (Table S1, see Appendix A1 for details).

Yet, distinguishing this cryptic diversity remains difficult as *L. minor* and *Lemna* × *japonica* hybrids are virtually morphologically indistinguishable (Smith et al. 2025; Volkova et al. 2023) (although some differences have been detected in physiological traits, including light, water chemistry, and temperature preferences: Smith et al. 2025; Smith et al. 2023; Volkova et al. 2024). Importantly, they play more similar ecological roles to one another compared to the range‐shifter *S. polyrhiza* genotype (Couture et al. 2025; Jewell and Bell 2023b; Smith et al. 2025; Volkova et al. 2023). Moreover, their readily hybridizing behavior indicates that reproductive barriers have been lost (Ernst et al. 2025; Schmid et al. 2024), suggesting that hybrids potentially act as additional sources of intraspecific genetic diversity. Therefore, we consider genotypic sorting in the *Lemna* species complex to be reasonably representative of the intraspecific genotypic sorting underlying evolution and will henceforth refer to confirmed *L. minor* , *Lemna* × *japonica*, and unconfirmed genotypes as *L. minor* for convenience.

### 2.2 Duckweed Preparation

We bleached all strains of *L. minor* and *S. polyrhiza* to eliminate algal, fungal, and bacterial contamination, following a protocol by Usui (2023). Following bleaching, we grew fronds under conditions that facilitate rapid growth: *N* (Appenroth) media (Appenroth et al. 1996), high light (under 30 W LED Sublaster T5 fluorescent grow lights) with a 16:8 h light‐to‐dark ratio, and at room temperature (~21°C). Once each genotype had multiplied sufficiently, we transferred the fronds to lower‐nutrient conditions for ~10 days in a different medium that simulates the nutrient composition of a pond (D‐media; Docauer 1983). We transitioned fronds in this way, from 100% *N*‐media to 10% D‐media (before growth in 5% D‐media during the experiment), to limit nutrient storage which could cause unwanted overshooting of population equilibrium densities. Such nutrient storage has been observed in duckweeds, particularly with phosphorus, which can affect growth trajectories for several weeks following nutrient transition (Peeters et al. 2016). Overshooting the expected equilibrium densities would have complicated our method for detecting any rapid evolution in *L. minor* populations, as described below.

### 2.3 Sustained Range Expansion Experiment

Fifty populations of *L. minor* consisting of the eight genotypes in Figure 1 were constructed at approximately equal relative abundances (12% ± 2%) between June 5–12, 2023, in 9 cm diameter (8 oz) plastic cups with a target abundance of ~560 healthy fronds per cup (~70 fronds per genotype). We estimated this target by averaging isogenic population equilibrium densities on low‐nutrient media across all genotypes to estimate a pseudo‐equilibrium density before introducing the range‐shifter (Appendix A2, Figure S1). Each cup contained 100 mL of low‐nutrient, pond‐simulating media (5% Docauer media or D‐media), diluted with autoclaved, de‐ionized water (Docauer 1983). In preliminary trials, we found that this nutrient concentration was optimal for limiting significant frond overlap without hindering all vegetative growth or preventing *S. polyrhiza* growth. Limiting *L. minor* frond overlap, particularly once populations reached an equilibrium density, was a crucial component of this study design to allow us to visually detect changes in genotype composition.

To do so, we visually tracked changes in the relative abundance of *L. minor* genotypes during the experiment using unique colors of Sally Hansen Insta‐dri nail polish. We used toothpicks to apply small dots of nail polish to the center of fronds (Usui 2023; Appendix A3). During preliminary trials, we found evidence of nail polish toxicity as dot application significantly decreased population growth from undotted controls, but this effect did not significantly vary with nail polish color (Table S2, Figure S2). We nonetheless randomized the color assigned to *L. minor* genotypes for each replicate to prevent any persistent confounding effect of nail polish color on relative abundance between treatments.

Once the *L. minor* populations were assembled, we exposed half of them to interspecific competition with *S. polyrhiza* (exposed treatment) by introducing three randomly selected *S. polyrhiza* propagules into each cup, while the other half experienced only intraspecific competition among *L. minor* genotypes (unexposed treatment). The *S. polyrhiza* propagules consisted of three rafts, containing between 6 and 11 fronds (mean = 7.3). We did not mark the fronds of *S. polyrhiza* with nail polish dots, as they could be easily distinguished from *L. minor* . Ideally, the assembled *L. minor* populations would have been allowed to equilibrate to a polygenic equilibrium before introducing *S. polyrhiza* to further mimic a resident‐range expander scenario. However, we avoided this to reduce the duration for which *L. minor* was tracked with nail polish, thereby reducing potential error propagation in genotype tracking.

All populations (*N* = 50, 25 per treatment) were placed in a Conviron TC16 incubator on a 16:8‐h day‐to‐night cycle at 27°C (25°C at night), illuminated by Sunblaster T5 fluorescent grow lights. This high temperature served two purposes: (1) to approximate future, warmer temperatures that may be experienced at the locations from which these *L. minor* accessions were selected, and (2) to maximize the potential for *S. polyrhiza* to maintain positive growth rates as temperatures in this range appeared to maximize coexistence potential between the two species as measured by Armitage and Jones (2019). A temperature of 26°C is closer to the maximum rather than mean summer temperatures predicted for *L. minor* 's current range edge in Northern Europe by the end of the century under the most extreme climate change scenario (European Environment Agency 2026).

We grouped unexposed and exposed (to *S. polyrhiza* ) cups in replicate blocks and evenly distributed these blocks among and within incubator racks. We often and haphazardly mixed the positions of these blocks in the incubator. The cups were all enclosed by domed lids with a 2.5‐cm‐diameter hole. This hole was left unblocked to allow nail polish fumes to escape and to prevent excess humidity build‐up that could promote mold growth.

The experimental populations grew for 14 weeks (ending on September 6th, 2023). Over this period, we estimate that 8–10 generations (new fronds) were produced. We maintained the populations by (1) refreshing the nutrient media to maintain approximately chemostatic conditions, (2) dotting new clonal daughter fronds that arose from parental fronds with nail polish and occasionally re‐dotting fading markers, (3) periodically introducing new *S. polyrhiza* propagules to maintain a competitive selection pressure as *S. polyrhiza* did not perform as well as expected under the low nutrient conditions required for *L. minor* tracking, and (4) and performing mold mitigation measures. (See Appendix A4 for details on maintenance procedures). Despite attempts to mitigate mold growth, we chose to discard 5 cups (2 unexposed, 3 exposed) with substantial mold accumulation (Appendix A4).

### 2.4 Quantifying Resident Evolution & Plasticity in Response to Sustained Range Expansion Exposure

We photographed the surface of each cup weekly with a Canon Rebel T3i DSLR (18 MP) starting on June 7, 2023. We used the cell counter tool in ImageJ to manually count and estimate initial and final genotypic abundances, respectively from images for the populations without extensive mold presence and those not subject to accidental physical disturbance remaining in the final week (see Appendix A4 for details). We counted all visible fronds at the surface, which accounted for the majority of fronds. Some live submerged fronds may not have been represented in the final week counts, but we assumed their contribution would be negligible, as frond overlap was minimal in the final week, and submerged fronds appeared to perish soon after submersion under these conditions. We used these counts to determine the temporal trend in the relative abundance of *L. minor* genotypes (RA, %) between the first and last weeks in both treatments: ∆RA = (N_*genotype*_/N_*total*_)_*week*14_ − (N_*genotype*_/N_*total*_)_*week*1_. We also quantified final week total *L. minor* abundance from these counts, the total surface area occupied by duckweeds (see Appendix A5 for methods), the total number of *S. polyrhiza* fronds added to exposed populations during the experiment, and qualitatively estimated mold extent in the final week (discrete covariate: 0 = not present, 1 = present, only on cup walls, 2 = larger patches present).

All analyzes were conducted in R version 4.2.1 (R Core Team 2022). We first compared the final week abundance and occupied surface area between treatments using Welch's *t*‐tests and *F*‐tests. Next, to determine whether evolution occurred in the resident due to *S. polyrhiza* exposure (Q1a), we used a simple linear model. The response variable was the temporal change in relative abundance (∆RA) of *L. minor* genotypes, and explanatory variables were treatment, genotype, and their interaction. We initially included cup replicate (pair/block) number as a random effect to account for variation between replicate blocks (Millar and Anderson 2004); however, this model had a singular fit. Therefore, we treated replicate as a fixed effect as recommended by Oberpriller et al. (2022). We removed twelve extreme outliers (likely due to errors in genotype tracking) based on a conservative Cook's Distance threshold (7/the sample size, 328), which represented a natural break in the data (Figure S3, Appendix S6). We used the “emmeans” package (Lenth 2022) to extract estimated marginal means for this model, to perform post hoc pairwise contrasts in ∆RA between treatments for each genotype, and to calculate selection imposed by exposure to the range‐shifter (henceforth, exposure‐driven selection) for the later analyzes: ∆RA_Exposed_ − ∆RA_Unexposed_, that is, the difference in estimated marginal means. This metric controls for temporal change in RA driven by intraspecific dynamics in unexposed populations.

We explored whether including the covariate of final week mold extent improved model fit using ANOVA comparisons. We further performed a sensitivity analysis of the original model (without the additional covariates), where we randomly swapped genotype identities within groups based on visual phenotypic similarity (group 1: L‐8623, L‐5500, L‐5576, L‐9495; group 2: L‐8784, L‐7436, L‐8625). We used error rates of 1%–10% error to mimic nail polish dotting mistakes, and we compared the average results across 999 iterations. Additionally, due to concerns of limited statistical power, we supplemented the first model for rapid evolution with a simpler alternative: a linear regression with genotype directly predicting potential differences in ∆RA_Exposed‐Unexposed_ between paired replicates remaining in week 14 (where neither cup in the pair was removed from the incubator due to extensive mold: see Appendix A4). Lastly, we used a simple linear model to explore whether varying quantities of *S. polyrhiza* fronds added to exposed populations affected ∆RA_Exposed‐Unexposed_, and whether this differed by *L. minor* genotype.

To determine whether simulated range‐shifter exposure induced morphological plasticity in resident genotypes (Q1b), we began by measuring four simple traits of sampled *L. minor* individuals from a sample of the experimental populations (*n* = 9) between September 6–11, 2023 (weeks 14–15 of the experiment) (Appendix A7). We measured(1) root length of the longest root on a raft (*N* = 447, 19–39 per treatment and genotype), (2) the number of live (green) fronds per raft (i.e., raft size) (*N* = 490, 24–40), (3) average frond area (*N* = 478, 21–40), and (4) root length/frond area (*N* = 470, 21–40). These samples were uneven as a by‐product of minimizing misidentification errors (see Appendix A7 for measurement details). To draw comparisons between treatments for these traits (i.e., to determine whether exposure induced morphological plasticity), we developed linear mixed models using the “lme4”, “lmerTest”, “performance”, and “DHARMa” packages (Bates et al. 2015; Kuznetsova et al. 2017; Hartig 2022; Lüdecke et al. 2021) (Appendix A7). Each trait was first predicted by treatment (unexposed or exposed), genotype, and their interaction, with cup replicate number as a random variable. If diagnostic graphs identified outliers, we applied a Cook's distance threshold of 4 divided by the sample size, recommended by Van der Meer et al. (2010) (e.g., root length). We first regressed raft size as a Poisson family model for count data; however, these data were significantly under‐dispersed, so we used a quasi‐Poisson family model, which better handles this scenario and produced smaller standard errors around model estimates (Zeviani et al. 2014). Additionally, we treated cup replicate as a fixed effect for raft size as the linear mixed model had a singular fit (Oberpriller et al. 2022).

### 2.5 Investigating Inter‐ and Intraspecific Selection on Resident Functional Traits

As duckweeds exhibit extensive phenotypic plasticity in response to their environment, particularly nutrient availability (Jewell and Bell 2023a; Peeters et al. 2016), we measured genotype‐specific functional trait means and variation in different nutrient and competitive conditions to generate predictors that could potentially explain any observed selection from the sustained range expansion experiment (Q2). In addition to the morphological trait measurements collected in section 2.3, we collected data on these same traits (root length, number of fronds per raft, average frond area, root length: frond area) for each *L. minor* genotype sampled from isogenic populations grown on high (100% D‐media) and low (5% D‐media) nutrient levels alongside the experimental populations (Appendix A8). We selected these nutrient conditions to reflect morphology relevant to the beginning and endpoints of the experiment, respectively, as generations became increasingly removed from the high‐nutrient media in which they were grown prior to the experiment. As low‐density per capita growth rates (i.e., the finite rate of increase) can be indicative of relative competitive ability (Aarssen 1983), we separately monitored the growth of two rafts seeded in small plastic cups under a single low‐nutrient condition, the same nutrient concentration used in the sustained range expansion experiment, to quantify competitive potential in such an environment (Appendix A8). We therefore calculated the mean per capita growth rate under low nutrients and low density (λ_L_) as the number of *L. minor* fronds present after 5 days divided by the number initially seeded (*n* = 5/genotype).

We first explored whether these traits significantly varied between genotypes within a treatment using a series of linear mixed and generalized linear models (Appendix A9). Next, to build a set of functional trait predictors for selection, we calculated baseline morphological trait means from the low and high‐nutrient isogenic samples. We also calculated plasticity for each morphological trait as the coefficient of variation across all sampled individuals from the isogenic and polygenic (experimental unexposed and exposed) populations (Valladares et al. 2006). This measure could indicate the ability to respond to a changing neighborhood of competitors and nutrient conditions, which could have arisen in this experiment. The final set of nine predictors included baseline trait means for root length, raft size, frond area, and root length‐to‐frond area ratio, and λ_L_, as well as estimates of plasticity for the morphological traits. We regressed each of these variables with model‐predicted exposure‐driven (∆RA_*Exposed‐Unexposed*_) and intraspecific competition‐driven (∆RA_*Unexposed*_) selection on *L. minor* genotypes observed in the sustained range expansion experiment.

### 2.6 Re‐Exposure Experiment

To determine if resident biotic resistance was affected by prior exposure history in the sustained range expansion experiment (Q3), we conducted a second experiment beginning on September 9, 2023, 1 day after the first experiment concluded, in which we challenged previously unexposed and previously exposed resident populations with new propagules of *S. polyrhiza* and compared *S. polyrhiza* growth rates between treatments (Appendix A10). We began by carefully transferring all *L. minor* fronds from the remaining populations not subject to trait sampling or extensive mold (*n*_unexposed_ = 19, *n*_exposed_ = 18) to new cups with fresh nutrient media at the same concentration established at the beginning of the first experiment. This was done in an attempt to limit the spread of mold growing on dead fronds on cup walls to the live frond surfaces, where it could more directly interfere with duckweed growth. We removed old propagules of *S. polyrhiza* from the previous experiment and exposed all populations to three new rafts of *S. polyrhiza* grown on 5% D‐media for > 1 month.

We recorded the abundance of *S. polyrhiza* at 15 days (Week 2) and 21 days (Week 3) to capture multiple potential generations of growth. For the response, we calculated the re‐exposure finite growth rate (λ_RE_) of *S. polyrhiza* as the final divided by the initial abundance. Under environmentally stressful conditions, which our experiment imposed, *S. polyrhiza* produces specialized dormant fronds (turions), which could invade differently from typical fronds (Appenroth et al. 1996; Docauer 1983; Jacobs 1947). In addition to total re‐exposure growth rates (λ_RE_) of all life stages, we therefore individually calculated re‐exposure growth rates for the following life stages: (1) surface (vegetative) fronds, (2) floating turions (juvenile or germinating turions), and (3) submerged turions (mature turions sunken to the bottom). We set the initial abundance to 1 for mathematical feasibility, both for floating and submerged turions, since we did not initially introduce any turions. We additionally noted for each replicate whether mold was visibly present in Week 1 or Week 3 (binary indices) and quantified the extent of mold during Week 1 as a qualitative index ranging from 1 to 10 (10–100% cup surface area). We removed two unexposed cups with substantial areas of necrotic fronds from mold accumulation from the analysis, leaving final sample sizes of *n*_unexposed_ = 17 and *n*_exposed_ = 18.

To determine whether prior exposure increased biotic resistance, we fit linear mixed models where each population growth metric was predicted by an interaction between time (categorical: Week 2, Week 3) and exposure history, as well as a fixed covariate, mold presence in Week 3 (binary) and *Lemna minor* population size as a random effect. We additionally created similar models swapping population size for mold extent during Week 1 as the random effect to capture potential confounding mold effects at both the beginning and the end of this experiment. For this model iteration, the vegetative frond growth model yielded a singular fit, so we instead fit a simple linear model with mold presence in Week 1 or Week 3 as a fixed effect.

## 3 Results

### 3.1 Q1: Rapid Evolution & Plasticity

Populations grew over time, reaching approximate equilibrium densities between Weeks 7–10 in unexposed populations and Weeks 8–13 in exposed populations (Figure S4). Unexposed and exposed populations did not differ in final week densities of *L. minor* (mean unexposed = 815 fronds ±SD 102, mean exposed = 864 ±SD 66 fronds; unpaired *t*‐test comparison of means *t*(39) = −1.80, *p* = 0.079; *F*‐test comparison of variability *F*(19, 20) = 2.39, *p* = 0.059) or total cup surface area occupied by duckweeds (mean unexposed = 12.6 cm^2^, mean exposed = 13.2 cm^2^, paired *t*‐test *t*(16) = 1.01, *p* = 0.32; *F*(16, 16) = 1.68, *p* = 0.31). Mold was present in 46% of replicates with relatively even presence across treatments (unexposed: 43% of replicates affected, exposed: 50% of replicates affected) (Table S4). Twelve extreme outliers with evidence of being the result of genotype‐tracking errors were identified and excluded from analyzes (but see Appendix B1, Tables S8 and S9).

**FIGURE 2:**
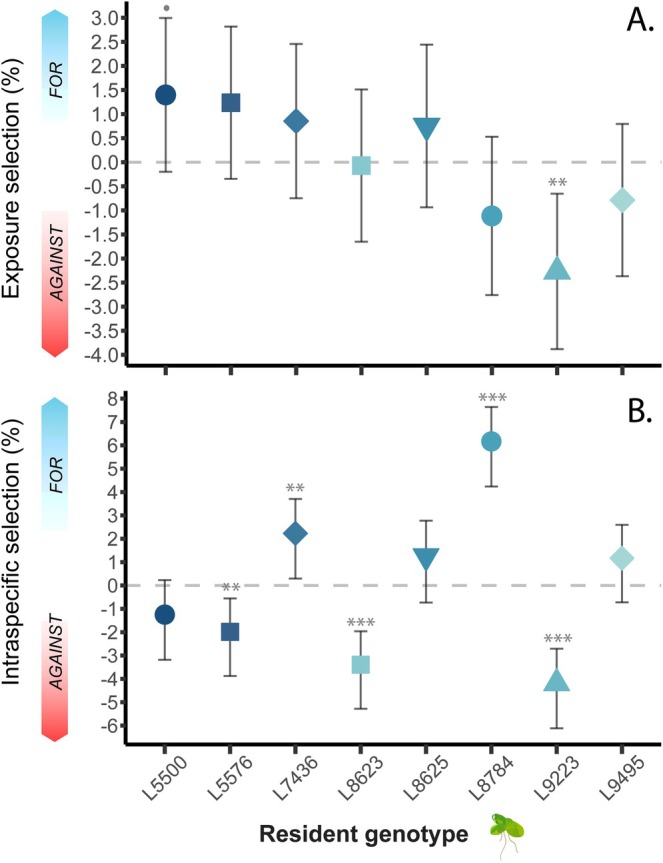
Predicted exposure‐driven selection (A) and intraspecific selection (B) on eight resident *Lemna minor* genotypes (±95% Tukey‐adjusted confidence intervals). Exposure‐driven selection was calculated as the difference in estimated marginal means of the change in relative abundance over 14 weeks (∆RA = RA_*week*14_—RA_*week*0_ (%)) between treatments, where populations were either exposed to low densities of a competitor duckweed, *Spirodela polyrhiza* (*n* = 20), or unexposed (*n* = 21) (exposure‐driven selection % = ∆RA_*Exposed*_ − ∆RA_*Unexposed*_). Intraspecific selection is simply the change in relative abundance over 14 weeks in the unexposed populations (i.e., sorting due to intraspecific competition). Positive values indicate selection for and negative values selection against particular genotypes. Values in both panels were scaled to sum to zero. *p* < 0.1 = “.”, *p* < 0.01 = **, *p* < 0.001 = ***. (*N* = 320, *n*_unexposed_ = 162, *n*_exposed_ = 154).

Some resident *L. minor* genotypes exhibited subtle differences in relative abundance trajectories between exposed and unexposed treatments, signaling significant exposure‐driven selection (∆RA_*Exposed*−*Unexposed*_) and rapid evolution (ANOVA treatment‐by‐genotype interaction *p* = 0.014) (Figure 2A, Tables S5–S7). These results were insensitive to genotype identification errors (Figure S5). One resident genotype decreased in both treatments, but did so more rapidly when exposed to *S. polyrhiza* (L‐9223: ∆RA_*Exposed*−*Unexposed*_ = −2.27%, *p* = 0.006, *n*_unexposed_ = 21, *n*_exposed_ = 20), while another increased in the presence of *S. polyrhiza* (L‐5500: ∆RA_*Exposed*−*Unexposed*_ = 1.40%, *p* = 0.08). A simpler alternative model, including only paired replicates, predicted similar changes in genotypic composition but provided stronger statistical support for an exposure‐driven evolution effect (ANOVA, *p* = 0.0022; Figure S6). Variation in the number of *S. polyrhiza* fronds introduced to exposed populations across replicates in order to sustain healthy *S. polyrhiza* propagules and selection pressure did not significantly influence the magnitude of exposure‐driven selection on *L. minor* genotypes (Figure S7). Additionally, considering the extent of mold presence did not significantly improve model fit. Finally, there was no clear trend in exposure‐driven selection for or against hybrid *Lemna* × *japonica* genotypes (L‐8784, L‐8625). However, considering intraspecific competition alone, two of these three hybrids were significantly selected for, whereas only confirmed non‐hybrid *L. minor* genotypes were selected against, suggesting that hybrids were stronger competitors under the experimental conditions (Figure 2B, Appendix B2).

**FIGURE 3:**
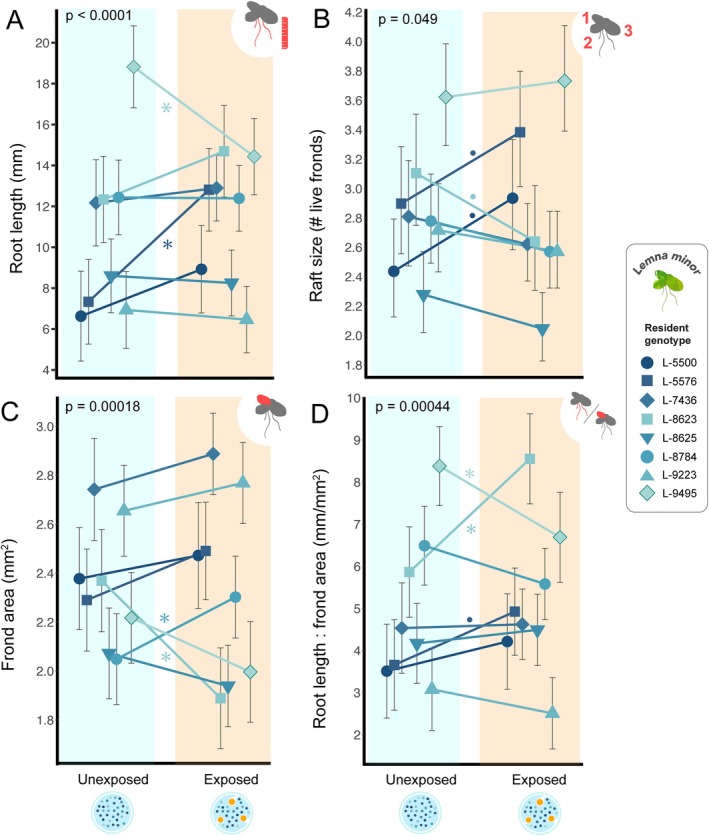
Estimated marginal means of four *Lemna minor* morphological traits (±95% CI) from linear models of trait predicted by an interaction between genotype and treatment (unexposed populations (blue) and populations exposed to low densities of *Spirodela polyrhiza* (orange)). (A) Root length (mm) (*n* = 22–40 rafts per treatment and genotype), (B) raft size (number of live fronds per raft) (*n* = 24–40), (C) frond area (mm^2^) (*n* = 21–40), and (D) root length: Raft area (mm/mm^2^) (*n* = 21–40) were measured after 15 weeks. Steeper lines connecting points indicate greater exposure‐driven trait plasticity. Asterisks denote significant post hoc pairwise contrasts between treatments (*p <* 0.05), and dots are marginally significant contrasts (*p* < 0.1). ANOVA *p*‐values for the treatment‐by‐genotype interaction are in the top left corners.

On average, resident genotypes did not display consistent directional plastic responses in morphology with *S. polyrhiza* exposure, but individual genotypes had significantly different root lengths, raft sizes, frond area, and root length: frond areas between treatments (Figure 3, Tables S10 and S11) (root length, *n* = 22–40: treatment × genotype *p* < 0.001; raft size, *n* = 24–40: treatment × genotype *p* = 0.049; frond area, *n* = 21–40: treatment × genotype *p* = 0.00018; root length/frond area, *n* = 21–40: treatment × genotype *p* = 0.00044). Two genotypes (L‐5576 and L‐9495) exhibited significantly different root lengths in the exposed treatment by 5.48 mm longer (a 68.8% increase relative to the mean root length of unexposed individuals) (*p* = 0.0003; 95% CI: 2.6, 8.4 mm) and 4.39 mm shorter (a 23.6.2% decrease) (*p* = 0.002; 95% CI: −7.1, −1.7 mm) respectively (Figure 3A). Two genotypes tended to have more fronds per raft in exposed populations (L‐5500: 0.50 fronds or a 20.2% increase; *p* = 0.051; 95% CI: −0.0026, 1.00 and L‐5576: 0.48 fronds or a 16.6% increase; *p* = 0.075; 95% CI: −0.049, 1.02) and one genotype tended to have fewer fronds per raft (L‐8623: −0.47 fronds or a 14.9% decrease; *p* = 0.079; 95% CI: −0.98, 0.053), although these individual post hoc contrasts were marginally significant (Figure 3B). Additionally, one genotype, L‐8623, had significantly smaller frond area in exposed populations by 0.48 mm^2^ (a 20.1% decrease) (*p* = 0.0016; 95% CI: −0.77, −0.19 mm^2^) while another had larger frond area by 0.15 mm^2^ (L‐8784: a 12.4% increase; *p* = 0.047; 95% CI: 0.0004 mm^2^, 0.50 mm^2^) (Figure 3C). Differences in absolute root length and frond area, also arose, again with one genotype increasing in root length relative to frond area (L‐8623: 2.69 mm/mm^2^ or a 46.3% increase; *p* = 0.0007; 95% CI: 1.17 mm/mm^2^, 4.21 mm/mm^2^) and another decreasing by 1.69 mm/mm^2^ (L‐9495: a 20.2% decrease; *p* = 0.021; 95% CI: −3.1 mm/mm^2^, −0.26 mm/mm^2^) (Figure 3D). These results are qualitatively similar to those from models using even sample sizes via bootstrapping (Menchions 2024), although the particular genotypes that exhibit significant responses differ.

### 3.2 Q2: Selection on Resident Functional Traits

**FIGURE 4:**
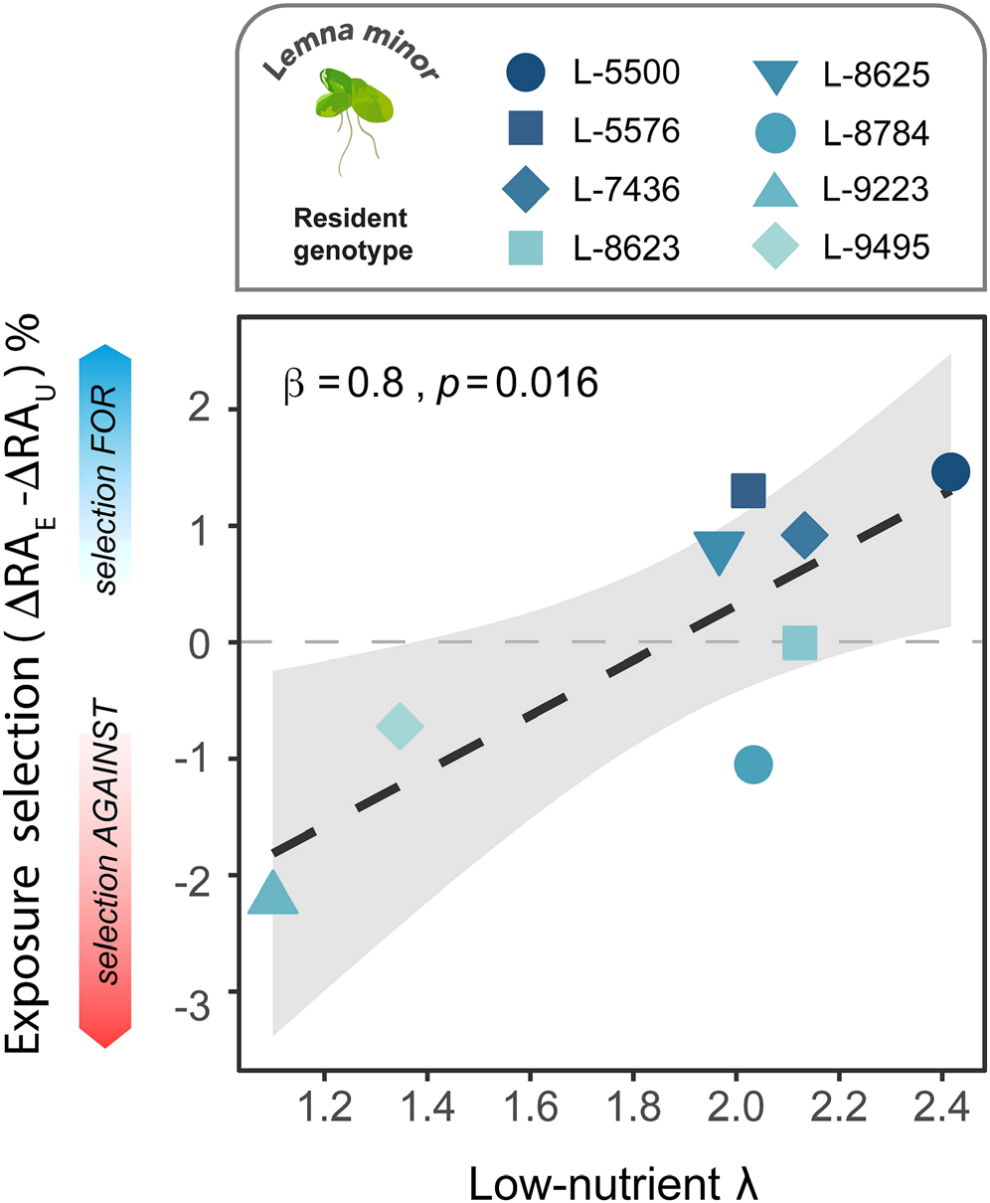
Exposure‐driven selection for growth rates of eight *Lemna minor* genotypes. Exposure‐driven selection is the difference in estimated marginal means for ∆RA (changes in relative abundance for *Lemna minor* resident genotypes over 14 weeks) between *L. minor* populations exposed to low densities of *Spirodela polyrhiza* and unexposed populations (exposure‐driven selection % = ∆RA_*Exposed*_ − ∆RA_*Unexposed*_). Low‐nutrient *λ* is the mean per capita growth rate under low nutrients (5% Docauer media) and low density, calculated as: Number of fronds_*day*5_/Number of fronds_*day*0_ across five replicates per genotype. Points represent genotypes, and the trendline is a simple linear regression (*β* = regression coefficient) bounded by 95% confidence interval bands (gray bands).

We generally found significant differences in root length, raft size, frond area, root: frond area, and λ_L_ among the *L. minor* genotypes selected for this experiment, although the extent of these differences varied with nutrient and competitive conditions (Tables S12 and S13). Despite these differences, exploratory analysis using a series of univariate linear regressions with these measured functional traits revealed that only one significantly explained some variation in exposure‐driven selection in our experiment: per capita growth rate under low nutrients and low density (λ_L_) (Figure 4, Table S14, Figure S8). Exposure‐driven selection was positively correlated with λ_L_ (*β* = 2.37). Multiple traits were also significantly correlated with selection driven by intraspecific competition (Appendix B2, Table S14, Figure S9).

### 3.3 Q3: Re‐Exposure

**FIGURE 5:**
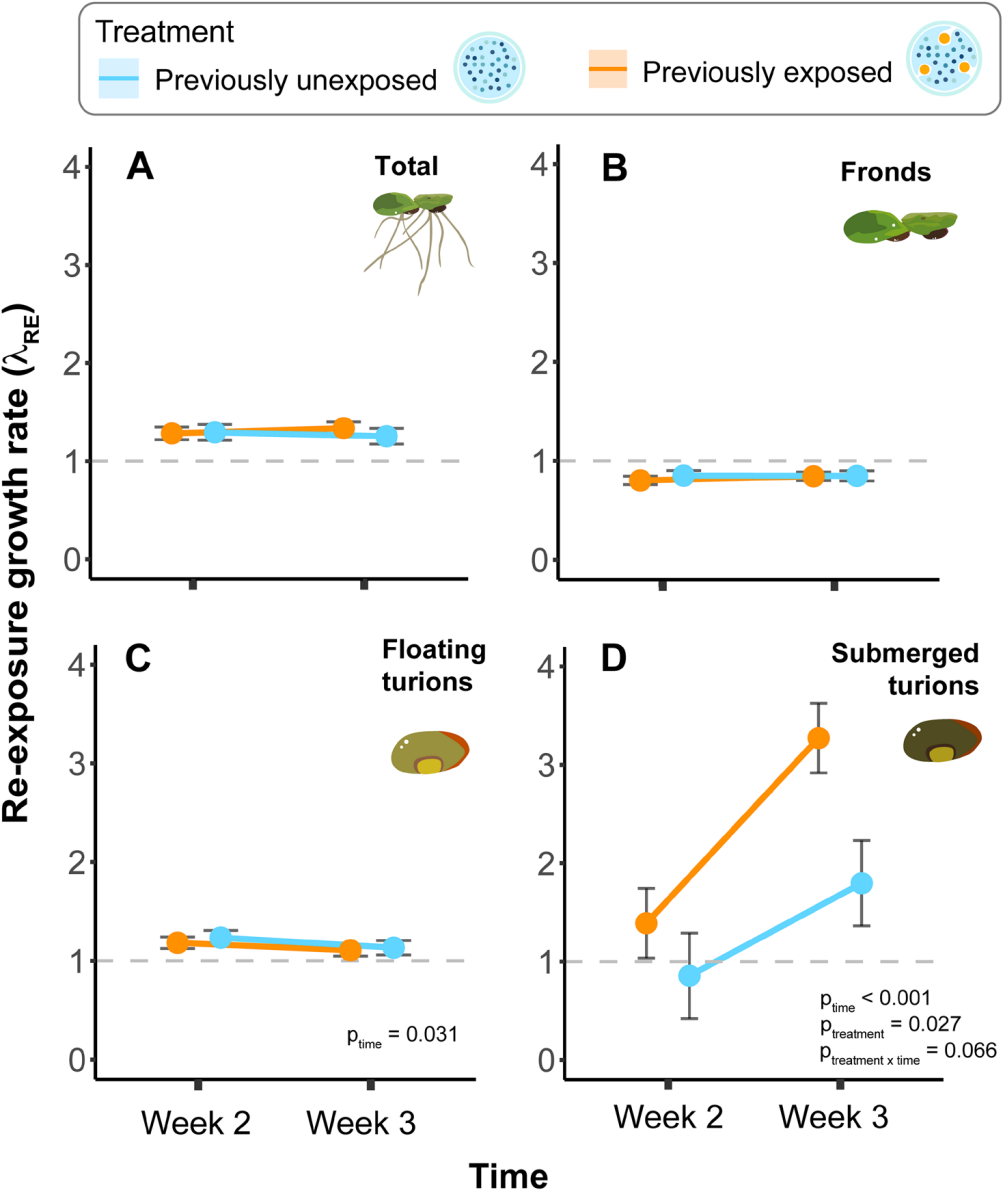
Estimated marginal means (points, ±SE) of finite re‐exposure growth rate (*λ*_*RE*_) of (A) total individuals, (B) fronds, (C) floating (juvenile) turions, and (D) submerged (mature) turions of the duckweed *Spirodela polyrhiza* from models with a treatment by time interaction (lines). Measurements were taken 15 days (week 2) and 21 days (week 3) after being reintroduced to previously exposed (orange, *n* = 17) and previously unexposed (blue, *n* = 18) (naïve) *Lemna minor* populations. *λ*_*RE*_ > 1 indicates that the abundance of a particular *S. polyrhiza* tissue increased, and *λ*_*RE*_ < 1 decreased. The underlying models used mold presence in week 3 (a binary variable) and *L. minor* population size as fixed and random effects, respectively. Significant ANOVA *p*‐values are displayed in the bottom right corner.

Despite efforts to reduce mold growth, mold was visibly present in some populations in the re‐exposure experiment and previously invaded populations were more affected (present in 50% of replicates) than previously uninvaded populations (29% of replicates). When pooling all *S. polyrhiza* life stages and accounting for this mold effect and population size, there were no significant differences in population growth rates over time between populations that were introduced to previously exposed versus previously unexposed *L. minor* resident populations (Figure 5, Tables S15 and S16). However, examining *S. polyrhiza* growth rates for different life stages separately, while accounting for mold effects and population size differences, revealed one interesting difference between treatments. Dormant tissue production in the form of turions increased over time (*p* < 0.0001) and was greater in the previously exposed populations (*p* = 0.0274, *n*_unexposed_ = 17, *n*_exposed_ = 18) (Figure 5D). These results were similar regardless of whether mold extent or population size was used as the random effect (Table S15).

## 4 Discussion

When prevalent, introduced species can induce evolutionary and plastic changes in resident communities that feed back to limit their own population growth. Here, we use a simulated range expansion to explore whether such biotic resistance could develop during the initial, low‐density expansion stages between species with an existing history of co‐occurrence in parts of their ranges, mimicking dynamics occurring during the early stages of asynchronous climate‐driven range shifts. Over 14 weeks of exposure to low densities of a simulated range‐shifting competitor at temperatures mimicking future climate change, we observed significant but subtle selection on (Figure 2) and morphological plasticity (Figure 3) in certain resident genotypes. This supports the hypothesis that range‐shifters may exert sufficient competitive pressure, even at low density, to drive rapid phenotypic changes in a resident species (Berthon 2015). We additionally found that, although exposure‐driven selection was not significantly positive for any particular genotype, the *L. minor* genotype with the fastest low‐nutrient growth rate numerically increased, and *L. minor* growth rates explained some variation in exposure‐driven selection generally (Figure 4). Finally, we observed some evidence for subtle changes in invasibility in previously exposed populations, even after accounting for the potentially confounding effect of mold contamination (Figure 5).

The significant exposure‐driven selection we observed is perhaps unexpected given the potentially weak competitive selective pressure exerted by *S. polyrhiza* at low densities. Although we detected significant exposure‐driven evolution, the rank order of genotypes did not change, and only one genotype showed a significantly different relative abundance in response to *S. polyrhiza* , indicating that *Lemna*'s rapid evolutionary response was correspondingly weak. Moreover, it is unknown how long selection would continue to act. Since the range‐shifter was constrained in its evolution (only a single genotype was added), it could not reciprocally evolve, so exposure‐driven selection pressure would have likely weakened as the resident reached a stable equilibrium genotype composition (Yamamichi et al. 2022).

There are a few potential explanations for the weak evolutionary response we observed. First, by establishing low‐nutrient conditions that allowed us to visually track changes in *L. minor* genotype frequencies, selection pressure from this abiotic stressor may have counteracted and/or masked the selection pressure exerted by *S. polyrhiza* . A second, non‐mutually exclusive explanation is that competitive pressure from *S. polyrhiza* was mostly experienced locally, resulting in weaker population‐level responses because the cups were not intentionally mechanically mixed during the experiment. This may suggest that the degree of selection on the resident in response to low invader densities depends on the movement capacity of the most limiting resource being competed for, as well as on the movement capacities of the resource and the interacting species. For instance, in this system, interference competition for space would likely only exert extremely local effects, whereas exploitation competition for aqueous nutrients could have had longer‐range (cup‐wide) effects. Following this reasoning, it is possible that local competition for space with *S. polyrhiza* played a key role, given that nearly 100% of the cup surface area was covered by duckweed fronds in mid‐late weeks of the experiment, though simultaneously, in higher‐nutrient conditions, duckweed populations are much denser (Laird and Barks 2018), and this axis of competition would likely be even more critical. Alternatively, if nutrient diffusion was low in the population cups, this may also have led to stronger local selection effects near *S. polyrhiza* propagules, especially since the growth media we used contained many macro‐ and micronutrients, offering multiple potential dimensions for competition (Docauer 1983; Usui 2023). Overall, however, we are unable to confirm the underlying competitive dynamics driving these observed changes in *Lemna*'s relative abundance. This is further complicated by *L. minor's* ability to produce allelopathic compounds that inhibit the growth of competitors (Gostyńska et al. 2022).

From some combination of these competitive mechanisms, we also observed significant exposure‐driven morphological plasticity, which, to our knowledge, has not been observed in response to low‐density introductions of a competitor, even in biological invasions (Berthon 2015). In a previous study, Hess et al. (2022) observed that a focal *L. minor* genotype developed greater root‐to‐shoot biomass following exposure to competition. Our results provide mixed support for this observation, with one genotype increasing (L‐8623) and another decreasing (L‐9495) in root length relative to frond area following exposure to *S. polyrhiza* . If these responses were adaptive despite directional differences, it is possible that the benefits of trade‐off strategies between light capture (associated with shoot allocation) and nutrient capture (associated with root allocation) varied across genotypes (Jewell and Bell 2023a). However, there is conflicting evidence about the importance of roots in nutrient uptake in *L. minor* (Cedergreen and Madsen 2002; Ware et al. 2023). Despite root length being the most readily plastic trait in natural populations along stress gradients (Jewell and Bell 2023a; Vasseur and Aarssen 1992), duckweeds can also uptake nutrients directly via frond surfaces and therefore frond area is also directly related to nutrient uptake, complicating inferences about differing root to shoot allocations (Cedergreen and Madsen 2002).

We also detected potential mixed plastic responses in raft size, with one genotype (L‐8623) numerically shifting to release daughter clones more slowly (i.e., larger raft sizes) following *S. polyrhiza* exposure, whereas others released clones more readily (L‐5500, L‐5576), although these individual contrasts were marginally significant. Again, if these responses were adaptive, it could suggest a trade‐off between competition in local space (i.e., delaying detachment to increase local competitive ability for light or space) and moving away from the parent to reduce competition (Bonte et al. 2012; Zhang et al. 2020). That said, these varied trait response directions are more likely to indicate a mixture of neutral, maladaptive, and adaptive plasticity (Turcotte and Levine 2016; Vasseur et al. 1993). Moreover, plasticity in most traits was not significantly associated with exposure‐driven selection on *Lemna* genotypes. However, these observations were limited to changes in morphological traits. Plasticity in many other unmeasured traits, including life history (e.g., generation time) and physiological traits (e.g., nutrient uptake rates), has been the most readily plastic in residents during biological invasions (Berthon 2015). Although unlikely, it is possible that unmeasured traits could have exhibited more consistent plastic responses to exposure, thereby differentiating the phenotypic composition between treatments and contributing to the observed treatment differences in the subsequent re‐exposure experiment.

Following exposure‐driven phenotypic changes in the resident, we observed increased production of submerged *S. polyrhiza* turions, even after accounting for potentially confounding effects of visible mold colonization and population size. This suggests that subtle phenotypic changes in the previously exposed resident populations influenced biotic resistance. Yet we are unable to definitively interpret the direction of this change in biotic resistance, as it depends on the temporal context under consideration. As turion production is a bet–hedging strategy, prioritizing long‐term over short‐term population growth through dormancy (Appenroth et al. 1996; Docauer 1983; Ellner 1987; Gremer and Venable 2014; Jacobs 1947), previously exposed resident populations may have been easier to invade as more turions in these populations may have maximized long‐term population persistence had conditions become favorable in the future. This interpretation suggests that the observed response reflected a decrease in biotic resistance (i.e., biotic accommodation). However, *S. polyrhiza* is known to produce more turions in response to abiotic and biotic stress for exmaple, competition (Strzałek et al. 2019). Similar responses have been observed in annual plants where rates of seed dormancy increase in competitively stressful or crowded communities in the short term (Ellner 1987; Gremer and Venable 2014). Therefore, an alternative interpretation is that the greater production in previously exposed populations we observed reflects *L. minor* populations exerting greater competitive pressure (i.e., greater biotic resistance), creating a more stressful environment in which turion production was more metabolically efficient than maintaining fronds for *S. polyrhiza* . This effect would be consistent with increased biotic resistance observed by Germain et al. (2020) in grasslands and Faillace and Morin (2016) in microbial communities. Overall, given the short time frame of our experiment and the lack of simulated environmental changes that could favor long‐term dormancy benefits, we are unable to determine the direction of the change in biotic resistance.

Regardless of direction, the subtle change in biotic resistance may have been driven by changes in genotypic relative abundances (direct effects) and/or traits (indirect effects) (Levine et al. 2017). Biotic accommodation could have arisen through selection for *L. minor* genotypes with greater niche differentiation with *S. polyrhiza* , adaptive plasticity in traits conferring greater niche differentiation, or selection for traits that decrease competitive ability (Hart et al. 2019). Alternatively, increased biotic resistance could have arisen in our system through selection for *L. minor* genotypes with traits conferring greater competitive ability relative to *S. polyrhiza* (e.g., Germain et al. (2020), Ferrero‐Serrano et al. (2011)), adaptive plasticity in similar traits toward greater competitive ability, and plasticity in traits toward increasing niche overlap with *S. polyrhiza* .

The only measured trait that appeared to be correlated with *L. minor* selection in response to *S. polyrhiza* exposure was low‐nutrient growth rate, suggesting selection for greater resident competitive ability (Aarssen 1983). Evolution along this axis of coexistence may be prevalent when opportunities for niche differentiation are limited (Abrams 1986). This observation may support our prediction of increased biotic resistance in response to exposure, but it could also be correlated with other traits related to niche differentiation, thereby supporting biotic accommodation (Levine et al. 2025). Additionally, although the directions of morphological plasticity we observed in *L. minor* in response to exposure were varied, if the individual responses were mostly adaptive, this could also have increased the relative competitive ability of particular genotypes or decreased niche overlap with *S. polyrhiza* , increasing overall resident fitness (Turcotte and Levine 2016). Conversely, these responses could have been mostly maladaptive, increasing niche overlap or decreasing relative competitive ability relative to *S. polyrhiza* . Yet, plastic responses are not necessarily adaptive or maladaptive, and we did not find evidence of selection for or against more morphologically plastic genotypes. We were unable to determine the relative contributions of selection and plasticity or the underlying mechanisms of each to the effects on biotic resistance following re‐exposure without additional treatments and reciprocal invasion experiments (Godwin et al. 2020; Hart et al. 2019).

The simplicity of this system allows us to disentangle processes that are difficult to detect in the field. However, our simulated range shift is not representative of the natural context of such a scenario for these species for three main reasons. (1) We did not use a *S. polyrhiza* genotype from Europe, but instead from North America. Although global genetic variation in *S. polyrhiza* is low (Ho et al. 2019; Xu et al. 2019) and plasticity contributes to much of their morphological variation (Jewell and Bell 2023a), phenotypic differences in our North American *S. polyrhiza* genotype from European genotypes may have elevated the novelty of the competitive interaction beyond what *L. minor* would experience from naturally range‐shifting *S. polyrhiza* . (2) By using novel combinations of resident genotypes rather than sampling populations in situ from near and beyond the range edge and given broad spatial distribution in the origins of our selected accessions we likely created *L. minor* populations with inflated genotypic diversity and ability to evolve (Schmid et al. 2024). (3) Finally, in an attempt to allow this strain of *S. polyrhiza* to invade under low‐nutrient conditions, the temperature at which we simulated range expansion exceeded the temperature increases expected during the growing season with climate change in Northern Europe, by ~5°C, even under the most extreme scenarios (Climate Impact Lab 2022; European Environment Agency 2026).

For these reasons, future work should aim to determine whether the patterns we observed here are possible and consequential in a natural context, including examining whether they are consistent across different genotypic compositions, species with more complex life histories, between residents and range‐shifters with varying extents of sympatry or parapatry, and in the presence of more complex species interactions and spatial dynamics. For instance, testing these methods on different resident genotypes would help clarify whether the relationship between low‐nutrient growth rate and exposure‐driven selection is a general pattern or specific to this sample of genotypes. Additionally, as many duckweeds have comparatively simple life histories and genomics (Laird and Barks 2018), it would be relevant to investigate whether higher mutation and recombination rates could dampen or accelerate any rapid local evolution in response to low competitor densities (North et al. 2011). In natural populations, dispersal between exposed and unexposed populations could also potentially weaken resident adaptation to sporadic range‐shifter exposures (Strauss et al. 2006; Vermeij 1982). Further, natural communities contain many interacting species, leading to complex outcomes, which our pairwise approach lacks (Levine et al. 2017; Strauss 2014). Therefore, subsequent work could involve natural experiments, assessing phenotypic and genotypic changes in multi‐species resident communities in response to low‐density colonization events along a climatic gradient, such as simulating up‐slope range expansion of plants in alpine meadows (Alexander et al. 2015; Tito et al. 2020). These experiments could more explicitly model spatial dynamics and movement along a gradient, which may affect residents' responses. Finally, we believe it would be critical to further investigate the relationship between range‐shifter density and resident evolution by examining resident responses across varying range‐shifter densities. Determining whether there are thresholds at which range‐shifters substantially affect resident phenotypes, and how this may differ across species and habitat contexts, would greatly enhance our understanding of the evo‐evolutionary dynamics of competition relevant to many range‐expansion scenarios.

Our results demonstrate that a potential range‐shifter may drive significant yet subtle microevolutionary and plastic changes in a resident competitor during the initial, low‐density stages of range expansion. Furthermore, it may occur on a short timescale relevant to ecological dynamics. This also suggests that evolution in a resident may be influenced by the presence of a low‐density competitor, not just by intraspecific sorting among its genotypes. Ecologists have recently begun to incorporate well‐established evolutionary principles into ecological theory, yet exposure‐driven resident evolution has not been considered in the recently reworked framing of Modern Coexistence Theory with eco‐evolutionary dynamics (Yamamichi et al. 2022). It is feasible that small changes in invasibility as our results suggested are possible, could scale up to have substantial impacts on the range‐expansion rates of range‐shifters in strongly competitive or environmentally stressful environments. Therefore, the evolution of biotic resistance could add further complexity to range‐expansion forecasting (Duchenne et al. 2021; Lenoir et al. 2020; Miller et al. 2020). Without considering eco‐evolutionary dynamics in range shifts, such as changes in biotic resistance, we may fail to capture the subtleties of species' redistributional responses in a climate‐changing world.

## Author Contributions

**Emma Menchions:** conceptualization (equal), data curation (lead), formal analysis (equal), funding acquisition (supporting), investigation (lead), methodology (equal), project administration (equal), resources (supporting), validation (lead), visualization (lead), writing – original draft (equal), writing – review and editing (equal). **Amy Angert:** conceptualization (equal), data curation (supporting), formal analysis (equal), funding acquisition (lead), methodology (supporting), project administration (supporting), resources (lead), supervision (lead), visualization (supporting), writing – original draft (equal), writing – review and editing (equal).

## Funding

This work was supported by the Natural Sciences and Engineering Research Council of Canada, Canadian Graduate Scholarship (Master's program), Discovery Grant, University of British Columbia, Vladimir J. Krajina Prize in Plant Ecology, University of British Columbia, British Columbia Graduate Scholarship.

## Conflicts of Interest

The authors declare no conflicts of interest.

## Supporting information


**Figure S1:** Finding isogenic equilibrium density points for eight *Lemna minor* genotypes. The trend line represents generalized additive (GAM) models with a k = 4 smoothing parameter. The gray zone indicates 95% confidence intervals from the trend line. Boxes are the interquartile range, whiskers are the full range, and horizontal lines are medians of population growth rate (surface area spread, cm^2^ per day) values across four replicates at each sampling date.
**Figure S2:** Impacts of applying small dots (< ~40% area) of various shades of Sally Hansen Insta‐Dri nail polish on population growth (frond production) in the *Lemna minor* complex over 10 days (*n* = 3 replicates per treatment). The x‐axis indicates the slope of linear regressions (change in fronds produced per day) produced by the following mixed effects model: Ln(number of fronds) = time*treatment + (1 | replicate). “Control 1” and “Control 2” treatments refer to replicate populations where fronds were not dotted with nail polish (gray bars and open circles). The growth trials included two genotypes (one *Lemna minor* and one *Lemna* × *japonica* hybrid) collected from the Greater Vancouver Area, British Columbia, Canada. Dots indicate estimated marginal mean slope values, and bars represent 95% confidence intervals.
**Figure S3:** Influential outliers identified by Cook's distance for differences in *Lemna minor* genotype relative abundances over 14 weeks in unexposed populations and populations exposed to *Spirodela polyrhiza* . The y‐axis indicates Cook's distance values of each observation (index). Outliers above the threshold of 7/*N*, where *N* = 328 observations. (*N* = 328, *n*_unexposed_ = 168, *n*_exposed_ = 168).
**Figure S4:** Total abundance of *Lemna minor* in the first eight replicate populations composed of eight *L. minor* genotypes where half of the populations were exposed to *Spirodela polyrhiza* at low density (right) and the other half were left unexposed (left). The means across these replicates are plotted with thick lines (blue = unexposed populations, orange = exposed populations).
**Figure S5:** Sensitivity analysis of rapid evolution models for results generated with (a) a 1% genotype misidentification rate and (b) a 10% genotype misidentification rate averaged across 999 bootstrap replicates. *Lemna minor* genotype identities were randomly swapped within cup replicates and within visual similarity groups (group 1: L‐5500, L‐5576, L‐8623, L‐9495; group 2: L‐8625, L‐8784, L‐7436). The genotype L‐9223 was not included in these random misidentifications as it was visually distinct. Exposure‐driven selection (±95% Tukey‐adjusted confidence intervals) (y‐axis) was calculated as the difference in estimated marginal means of the change in relative abundance over 14 weeks (∆RA = RA_week14_—RA_week0_ (%)) between treatments, where populations were either exposed to low densities of a competitor duckweed, *Spirodela polyrhiza* (*n* = 20), or unexposed (*n* = 21) (exposure‐driven selection % = ∆RA_Exposed_ − ∆RA_Unexposed_). (*N* = 328, *n*_unexposed_ = 168, *n*_exposed_ = 168).
**Figure S6:** Alternative rapid evolution model. Predicted exposure‐driven selection on eight resident *Lemna minor* genotypes (±95% Tukey‐adjusted confidence intervals). Exposure‐driven selection was calculated as the difference in the change in relative abundance over 14 weeks (∆RA = RA_week14_—RA_week0_ (%)) between paired replicate populations either exposed to low densities of a competitor duckweed, *Spirodela polyrhiza*, or unexposed (exposure‐driven selection % = ∆RA_Exposed_ − ∆RA_Unexposed_). Positive values indicate selection for and negative values selection against particular genotypes. The ANOVA *p*‐value is in the upper‐right corner. (*N* = 136, *n*_replicates_ = 17).
**Figure S7:** Observed range‐shifter exposure‐driven selection (%) experienced for eight *Lemna minor* genotypes, with the total number of range‐shifter propagules ( *Spirodela polyrhiza* fronds) added to each replicate. The y‐axis is the difference in observed ΔRA (changes in relative abundance for *L. minor* resident genotypes over 14 weeks) between populations within the same replicate, either exposed to (E) and unexposed to (U) *S. polyrhiza* at low density (Exposure‐driven selection % = ΔRA_E_—ΔRA_U_). Positive values indicate directional exposure‐driven selection “for” (blue), and negative values indicate directional selection “against” (red). The x‐axis is the total number of *S. polyrhiza* fronds added to exposed populations during a 14‐week‐long sustained range expansion experiment, including the initial number of fronds and the additional fronds added at weeks 7, 9, and 12. The trendlines are simple linear regressions (*β* = regression coefficient) bounded by 95% confidence interval bands (gray bands). (*N* = 136, *n*_replicates_ = 17).
**Figure S8:** Exploratory univariate linear regressions (black lines, *β* = regression coefficient) of model‐derived exposure‐driven selection (%) experienced by eight *Lemna minor* (resident) genotypes in polygenic cultures with mean trait values of sampled rafts across various nutrient and competitive environments. The y‐axis is the difference in ΔRA (changes in relative abundance of over 14 weeks) between *L. minor* populations exposed to (E) or unexposed (U) to *Spirodela polyrhiza* at low density (exposure‐driven selection % = ΔRA_E_ − ΔRA_U_). “High nutrient” and “Low nutrient” indicate that trait means are derived from isogenic populations growing on high nutrient media or low nutrient media, respectively. λ = per capita low density growth rate, RL = longest root length of raft (mm), FA = average frond area (mm^2^), RS = raft size (number of fronds alive), RLFA = root length to frond area ratio (mm/mm^2^). Trait plasticity was calculated as the coefficient of variation in traits across *L. minor* rafts sampled from isogenic populations grown under high or low nutrient conditions, as well as polygenic populations exposed or unexposed to *Spirodela polyrhiza* at low density. The gray bands are 95% confidence intervals.
**Figure S9:** Exploratory univariate linear regressions (black lines, *β* = regression coefficient) of model‐derived intraspecific competition‐driven selection (%) experienced by eight *Lemna minor* (resident) genotypes in polygenic cultures with mean trait values of sampled rafts across various nutrient and competitive environments. The y‐axis is the change in relative abundance over 14 weeks in *L. minor* populations (intraspecific‐driven selection %, ΔRA_U_). “High nutrient” and “Low nutrient” indicate that trait means are derived from isogenic populations growing on high nutrient media or low nutrient media, respectively. λ = per capita low density growth rate, RL = longest root length of raft (mm), FA = average frond area (mm^2^), RS = raft size (number of fronds alive), RLFA = root length to frond area ratio (mm/mm^2^). Trait plasticity was calculated as the coefficient of variation in traits across *L. minor* rafts sampled from isogenic populations grown under high or low nutrient conditions, as well as polygenic populations exposed or unexposed to *Spirodela polyrhiza* at low density. The gray bands are 95% confidence intervals. Bolded axis titles indicate significant regressions (*p* < 0.05).
**Table S1:** Identities and collection locations of eight duckweed genotypes identified as *Lemna minor* by outdated genetic markers selected from the Rutgers Duckweed Stock Cooperative (RDSC). “Genotype” is the name we refer to each accession, whereas “Landolt ID” is the ID assigned by the Landolt collection. “Hybrid?” indicates which genotypes have recently been identified as hybrids between *Lemna minor* and *Lemna japonica* (Y = yes, *N* = no,? = has not yet been tested with updated genetic markers). Coordinates for each locality were georeferenced using Google Maps. ND = distance from the genotype accession location to the nearest occurrence of *Spirodela polyrhiza* (km). Coordinates for each locality were georeferenced using Google Maps.
**Table S2:** ANOVA summary of a mixed linear regression of the natural log of *Lemna minor* fronds present in a cup predicted by an interaction between day since the beginning of the experiment and color of Sally Hansen Insta‐Dri nail polish applied in small dots (< ~40% area) to frond surfaces (*n* = 3 replicates per treatment). The effect of color also included two undotted (control) treatments. Genotype (one *Lemna minor* and one *Lemna* × *japonica* hybrid) and replicate were treated as fixed and random effects, respectively. *p* < 0.005 = ***, *p* < 0.05 = *. (*N* = 173).
**Table S3:** Impacts of applying dots of one color of Sally Hansen Insta‐Dri nail polish (garnet) of varying sizes to *Lemna minor* fronds on population growth (frond production) over 10 days. Large dots (L) encompassed ~70% of frond surface area, whereas small dots (S) were applied to < ~40% frond area (*n* = 5). Fronds were left undotted in the control treatment (*n* = 10 replicates). Growth rate is the slope of regressions produced by the following linear model: Ln(Number of fronds) = time*treatment; indicative of the change in fronds produced per day. This growth trial included a single *L. minor* genotype collected from the Greater Vancouver Area, British Columbia, Canada. SE = standard error.
**Table S4:** Assessment of mold presence in week 14 of the remaining populations in the sustained range expansion experiment. All = all populations, U = unexposed *Lemna minor* populations, E = *L. minor* populations exposed to *Spirodela polyrhiza*. “Present” is the number of populations with visible patches of mold and the percentage out of all replicates remaining (all: *N* = 41, *n*_unexposed_ = 21, *n*_exposed_ = 20). “Only on walls” indicates when mold was present but not visibly growing over duckweed. “Large patches” were noted when mold was overgrowing > 2 duckweed rafts. The remaining columns denote the instances of mold of different colors. The percentages listed for columns 3 to 8 are relative to the number of populations where mold was present.
**Table S5:** ANOVA summary of a linear regression between temporal change in the relative abundance of eight *Lemna minor* duckweed genotypes and a treatment by genotype interaction with replicate number as a fixed effect (ΔRA = treatment*genotype + replicate), where treatment has two levels, unexposed or exposed to the duckweed *Spirodela polyrhiza* at low density. *p* < 0.005 = ***, *p* < 0.05 = *. (*N*_total_ = 316, *n*_exposed_ = 154, *n*_unexposed_ = 162).
**Table S6:** Estimated marginal means of linear regression slopes between temporal change in the relative abundance of eight *Lemna minor* duckweed genotypes (ΔRA) and a treatment by genotype interaction with replicate number as a fixed effect (ΔRA = treatment*genotype + replicate), where treatment has two levels, unexposed (U) or exposed (E) to the duckweed *Spirodela polyrhiza* at low density. SE = standard error, CI = confidence interval. *p*‐values were adjusted using the Bonferroni and Tukey methods with the “emmeans” R package, but 95% CI were presented only with the Bonferroni method because they were very similar across the adjustment methods. (*N*_total_ = 316, *n*_Exposed_ = 154, *n*_unexposed_ = 162). Estimates were scaled to add to 0 for each treatment, and the 95% CIs were also scaled accordingly.
**Table S7:** Pairwise post hoc contrasts between temporal changes in the relative abundance of eight *Lemna minor* genotypes over 14 weeks between populations exposed to (E) or unexposed to (U) *Spirodela polyrhiza* at low density (i.e., exposure selection % = ΔRA_E_—ΔRA_U_). Contrasts were performed using the emmeans “contrast” function, with family‐wise *p*‐values adjusted using the “mvt” method. Exposure selection estimates were scaled to add to 0. (*N*_total_ = 316, *n*_Exposed_ = 154, *n*_Unexposed_ = 162).
**Table S8:** Outliers identified by a Cook's distance threshold of 7/sample size for the model predicting temporal change in the relative abundance of eight *Lemna minor* duckweed genotypes over 14 weeks (ΔRA) with a treatment‐by‐genotype interaction and replicate number as a fixed effect (ΔRA = treatment*genotype + replicate). Treatment has two levels: unexposed (U) or exposed (E) to the duckweed *Spirodela polyrhiza* at low density. “Cup ID” corresponds to separate population cups with two per replicate block. “Nail polish color” corresponds to the color of nail polish applied in small dots to the surface of *L. minor* fronds. “Anomaly” is the deviation in ΔRA from the average for that genotype and treatment.
**Table S9:** (a) ANOVA summary and (b) pairwise post hoc contrasts of two alternative models of rapid evolution in *Lemna minor* upon sustained exposure to *Spirodela polyrhiza* over 14 weeks, varying by the outliers excluded. Model 1 excludes four paired outliers (two per cup) which may have been mistaken for each other in their respective replicates (*N* = 324). Model 2 excludes seven outliers that were dotted with nail polish colors that tended to be reapplied to fronds more than other colors, potentially inhibiting growth rates (*N* = 321). Both models were calibrated as simple linear regressions between temporal change in the relative abundance of eight *Lemna minor* duckweed genotypes and a treatment‐by‐genotype interaction with replicate number as a fixed effect (ΔRA = treatment × genotype + replicate), where treatment has two levels, unexposed or exposed to the duckweed *S. polyrhiza* at low density. Contrasts were performed using the emmeans “contrast” function, with family‐wise *p*‐values adjusted using the “tukey” method. *p* < 0.005 = ***, *p* < 0.05 = *. (*N* = 320).
**Table S10:** ANOVA results of morphological plasticity linear models where measured traits are predicted by an interaction between treatment (either unexposed “U” or exposed to “E” *Spirodela polyrhiza* at low density for 14 weeks) and *Lemna minor* genotype. The models in (A) were constructed as linear mixed models with cup replicate ID as a random factor (root length: *N* = 447, 22–40 per treatment and genotype; frond area: *N* = 478, 21–40; root length/frond area: *N* = 470, 21–40). Model (B) is a quasi‐Poisson family generalized linear model (*N* = 490, 24–40 per treatment and genotype). Bolded values indicate *p* < 0.05. Resid. = residual. df = degrees of freedom. Num = numerator. Den = denominator.
**Table S11:** Pairwise post hoc contrasts for linear models between sampled trait means of eight *Lemna minor* genotypes and treatment: unexposed populations (U) and populations exposed to *Spirodela polyrhiza* at low density (E) for 14 weeks. Contrasts were performed with the emmeans “contrast” function, and family‐wise *p*‐values were adjusted using the “mvt” method. SE = standard error, LCL = lower 95% confidence interval, UCL = upper 95% confidence level. *p*‐values are bolded for significant relationships (*p* < 0.05). Sample sizes are as follows: root length: *N* = 447, 22–40 per treatment and genotype; raft size: *N* = 490, 24–40; frond area: *N* = 478, 21–40; root length/frond area: *N* = 470, 21–40.
**Table S12:** ANOVA results of linear models comparing measured traits in *Lemna minor* among genotypes. Models in part (A) are derived from linear mixed models with an interaction between genotype and treatment, with cup replicate ID as a random factor. Treatments included isogenic + low‐nutrient, isogenic + high‐nutrient, polygenic + low‐nutrient, polygenic + low‐nutrient + exposure to the competitor *Spirodela polyrhiza* over 14 weeks (root length: *N* = 802, 20–40 per treatment and genotype; frond area: *N* = 798, 20–40; root length/frond area: *N* = 790, 20–40). The model from (B) was fitted with a quasi‐Poisson family generalized linear model with an interaction between treatment and genotype and cup replicate ID as a fixed factor (*N* = 810, 20–40). (C) Was modeled as a simple linear regression with genotype predicting low‐nutrient, low‐density growth rate, calculated as the number of fronds produced after five days divided by the initial number of fronds across two rafts in small cups (*N* = 40, 5 per genotype). Bolded values indicate *p* < 0.05. Resid. = residual. df = degrees of freedom. Num = numerator. Den = denominator.
**Table S13:** ANOVA results of linear models comparing measured traits between confirmed *Lemna minor* (L‐5500, L‐5576, L‐8263, L‐9495) and *Lemna* × *japonica* (L‐7436, L‐8625, L‐8784) genotypes. Models in part (A) are derived from linear mixed models with an interaction between hybrid status and treatment, with cup replicate ID as a random factor. Treatments included isogenic + low‐nutrient, isogenic + high‐nutrient, polygenic + low‐nutrient, polygenic + low‐nutrient + exposure to the competitor *Spirodela polyrhiza* over 14 weeks (Frond area: *N* = 686, 60–119 per treatment and hybrid status; Root length/frond area: *N* = 682, 60–118). The model from (B) was fitted with a quasi‐Poisson family generalized linear model (*N* = 698, 60–120). The models under section (C) are simple linear regressions (Root length: *N* = 60–119, λ_L_: *N* = 40, 5 per genotype). Bolded values indicate *p* < 0.05. Resid. = residual. df = degrees of freedom. Num = numerator. Den = denominator.
**Table S14:** Summary of linear regression results from univariate models between exposure‐driven selection or intraspecific selection and trait means and plasticities measured in *Lemna minor* genotypes (*N* = 8). Range shifter exposure‐driven selection is the difference in estimated marginal means for ΔRA (changes in relative abundance for *L. minor* resident genotypes over 14 weeks) between resident populations exposed to (E) and unexposed to (U) *Spirodela polyrhiza* at low density (Exposure selection % = ΔRA _E_—ΔRA _U_). Intraspecific selection is the change in estimated marginal means of relative abundance of *L. minor* genotypes over 14 weeks in populations unexposed to *S. polyrhiza* . λ = per capita low‐density, low‐nutrient growth rate; RL = root length (mm); FA = frond area (mm^2^); RS = raft size (count); RLFA = root length to frond area ratio (mm/mm^2^).
**Table S15:** ANOVA summaries of linear regression models of finite re‐exposure growth rate (λ_RE_) of *Spirodela polyrhiza* , fronds, floating (juvenile) turions, and submerged (mature) turions, 15 and 21 days after being introduced to previously unexposed/naïve (*n* = 18) and *Lemna minor* populations previously exposed to *S. polyrhiza* at low density (*n* = 17). λ_RE_ was calculated as the final divided by the initial abundance of each tissue type. Mold presence was quantified as visible mold presence (binary) in week 3 or weeks 1 and 3 of the experiment. In models labeled “M1”, mold extent (0–10) was used as a random covariate, while models labeled “M2” used *Lemna minor* population size in week 14 was used as a random variable. For floating and submerged turions, the initial abundance was set to 1 for mathematical feasibility. “df” = degrees of freedom. df = degrees of freedom. Num = numerator. Den = denominator. Model (A) is a simple linear regression, while models in (B) are linear mixed models.
**Table S16:** Summary of estimated marginal means of linear regression models of finite re‐exposure growth rate λ_RE_ of *Spirodela polyrhiza* , fronds, floating (juvenile) turions, and submerged (mature) turions, 15 and 21 days after being introduced to previously unexposed/naïve (U, *n* = 18) and *Lemna minor* populations previously exposed to *S. polyrhiza* at low density (E, *n* = 17). λ_RE_ was calculated as the final divided by the initial abundance of each tissue type. Each model treated mold presence in week 3 (binary) and *Lemna minor* population size in week 14 as fixed and random effects, respectively. For floating and submerged turions, the initial abundance was set to 1 for mathematical feasibility. “CI” = 95% confidence interval. “EMM” = estimated marginal mean (%). “df” = degrees of freedom.

**Data S1:** Supplementary Methods: Frond area estimation protocol.

## Data Availability

The data that support the findings of this study are openly available in Zenodo at http://doi.org/10.5281/zenodo.13733051.
