# Supplementary material for: Simulated Range Expansion Suggests Rapid Change in Biotic Resistance to a Range‐Shifting Competitor
Source: Ecol Evol. 2026 Jul 29;16(8):e74096. doi: 10.1002/ece3.74096 (PMC13420321; doi:10.1002/ece3.74096)
Supplement: Supplementary file 1 — Figure S1: Finding isogenic equilibrium density points for eight Lemna minor genotypes. The trend line represents generalized additive (GAM) models with a k = 4 smoothing parameter. The gray zone indicates 95% confidence intervals from the trend line. Boxes are the interquartile range, whiskers are the full range, and horizontal lines are medians of population growth rate (surface area spread, cm2 per day) values across four replicates at each sampling date. Figure S2: Impacts of applying small dots (< ~40% area) of various shades of Sally Hansen Insta‐Dri nail polish on population growth (frond production) in the Lemna minor complex over 10 days (n = 3 replicates per treatment). The x‐axis indicates the slope of linear regressions (change in fronds produced per day) produced by the following mixed effects model: Ln(number of fronds) = time*treatment + (1 | replicate). “Control 1” and “Control 2” treatments refer to replicate populations where fronds were not dotted with nail polish (gray bars and open circles). The growth trials included two genotypes (one Lemna minor and one Lemna × japonica hybrid) collected from the Greater Vancouver Area, British Columbia, Canada. Dots indicate estimated marginal mean slope values, and bars represent 95% confidence intervals. Figure S3: Influential outliers identified by Cook's distance for differences in Lemna minor genotype relative abundances over 14 weeks in unexposed populations and populations exposed to Spirodela polyrhiza . The y‐axis indicates Cook's distance values of each observation (index). Outliers above the threshold of 7/N, where N = 328 observations. (N = 328, n unexposed = 168, n exposed = 168). Figure S4: Total abundance of Lemna minor in the first eight replicate populations composed of eight L. minor genotypes where half of the populations were exposed to Spirodela polyrhiza at low density (right) and the other half were left unexposed (left). The means across these replicates are plotted with thick lines ( [file ECE3-16-e74096-s002.docx]

**Simulated range expansion suggests rapid change in biotic resistance to a range-shifting competitor**

Appendix A. Supplementary Methods

**Appendix A1**. Resident genotype hybrids

**Appendix A2**. Estimating a target initial abundance

**Appendix A3.** Effects of nail polish dots on growth

**Appendix A4**. Sustained range expansion experiment maintenance

**Appendix A5**. Estimating final surface area occupied

**Appendix A6.** Rapid evolution model outlier analysis

**Appendix A7.** Measuring *L. minor* trait plasticity

**Appendix A8**. Measuring *L. minor* functional traits

**Appendix A9.** Exploring functional trait variation between *L. minor* genotypes

**Appendix A10.** Re-exposure experiment details

Appendix B. Supplementary results

**Appendix B1.** Rapid evolution model outlier assessment & alternative models

**Appendix B2.** Selection driven by intraspecific competition

Supplementary Figures

**Figure S1**. Isogenic resident populations reaching equilibrium

**Figure S2**. Nail polish dot size effects on *L. minor* growth

**Figure S3.** Nail polish colour effects on *L. minor* growth

**Figure S4.** Rapid evolution model influential outliers

**Figure S5.** Sustained range expansion experiment population growth

**Figure S6.** Exposure-driven selection with propagule pressure

**Figure S7.** Exposure-driven selection with functional traits (exploratory regressions)

**Figure S8.** Intraspecific-driven selection with functional traits (exploratory regressions)

Supplementary Tables

**Table S1**. Resident genotype information

**Table S2.** Assessment of mold presence in sustained range expansion experiment

**Table S3.** ANOVA summary of rapid evolution model

**Table S4.** Estimated marginal means of rapid evolution model

**Table S5.** Pairwise post-hoc contrast for rapid evolution model

**Table S6.** Plasticity model mean ANOVA summary across 5000 bootstrap runs

**Table S7.** Plasticity model mean pairwise contrasts across 5000 bootstrap runs

**Table S8.** Summary of functional trait selection linear regression models

**Table S9.** ANOVA summaries for re-exposre models

**Table S10.** Estimated marginal means for re-exposure models

**Appendix A. Supplementary Methods**

**A1. Resident genotype hybrids**

New genetic barcoding techniques have revealed cryptic diversity within *Lemna minor,* relevant to the genotypes we used in our experiments (Braglia et al., 2021a; Braglia et al., 2021b; Senevirathna et al., 2021). With the development of updated genetic markers (Tubulin-Based Polymorphism profiling) by Braglia et al. (2021a) and Braglia et al. (2021b), many genotypes previously attributed as *L. minor* based on morphological characteristics and older barcoding methods have been re-identified as hybrids. One of these cryptic hybrids is *Lemna x japonica*, a cross between *L. minor* and a sister species *L. turionifera.* Genetic evidence suggests that it has arisen multiple times in independent hybridization events (Braglia 2021a; Ernst et al., 2025; Schmid et al., 2024). *Lemna x japonica* is highly similar in morphology to both parent species (Volkova et al., 2023). It has recently been revealed to be more widespread than expected in Europe (Schmid et al., 2024; Smith et al., 2025) and more prevalent in eastern Europe than either parent species (Volkova et al., 2023).

Seven of the eight putative *L. minor* accessions we used in our study were included in reassessments with updated genetic markers by Braglia et al. (2021a, 2021b) and Michael et al. (2025). Four of these were reconfirmed as *L. minor* (L-5500, L-8623, L-5576, and L-9495), but another three were re-attributed as *Lemna x japonica* hybrids (L-8784, L-8625, L-7436) (table S1). Interestingly, however, the genotype L-8625 was first categorized as *L. minor* by Braglia et al. (2021b) and subsequently as *L. x japonica* by Braglia et al. (2021a), highlighting the complexity and difficulty of differentiating these hybrids. The remaining accession we used (L-9223) has not yet been assessed with these updated methods and appears dubious based on its traits. It has shorter roots than the other *L. minor* genotypes, which could indicate that it is a hybrid between *L. minor* and another species, *L. minuta,* introduced to Europe, as there is some evidence of hybridization between these species in the UK, where this accession was collected (Smith et al., 2025). However, we were unable to definitively determine its status without genomic methods beyond the scope of this study. In summary, distinguishing entities within this species complex is difficult because of their morphological and ecological similarities to one another, yet because they are all very distinct from *Spirodela polyrhiza*, we consider evolutionary sorting of these lineages to be representative of intraspecific evolution in response to interspecific competition.

**A2. Estimating a target total abundance for assembling resident populations in the sustained range expansion experiment**

To estimate a target total abundance at which to assemble *L. minor* (resident) populations for the sustained range expansion experiment, we measured isogenic equilibrium densities (K_isogenic_) for each *L. minor* genotype in the lab under conditions similar to those used in the main experiment. We set up four 8-oz cups containing 100 mL of 5% D-media and seeded each with ~100 fronds for each genotype until the water surface was mostly covered. We placed all cups (8 genotypes × 4 replicates) in the same incubator used for the sustained range expansion experiment, in a stratified random arrangement, for 2 weeks. Every 2-4 days, we added autoclaved deionized water to compensate for evaporation, and we refreshed the media (removing 10 mL of liquid and adding 10 mL of fresh 5% D-media). We also photographed the surface of cups with a ruler near the water surface as a reference scale. We first used these photos to calculate changes in the total surface area occupied by fronds with a customized macro script in ImageJ adapted from Usui (2023).

To determine when each genotype reached an approximate equilibrium at which the total water-surface area covered by fronds was stable, we used generalized additive models (GAMs) with a smoothing parameter k = 4. We plotted the fitted lines and identified the sampling date at which the confidence intervals of the model fit crossed the zero-population growth rate (cm^2^/day) intercept (a method also adapted from Usui (2023)). We chose day 12 (2023-05-29) when most genotypes met this criterion (figure S1). We digitally counted fronds from the images taken on that sampling date for all replicates and genotypes. We then calculated the average frond abundance across all replicates for each genotype and averaged these values across all genotypes. This yielded an abundance estimate of 585 fronds. In the main experiment, we slightly reduced the target population size to 560 to buffer against the possibility of transient overcrowding due to nutrient storage. Since this equilibrium value only represented an average of isogenic equilibria, and we set up our experimental populations with equal genotype frequencies (560/8 = 70 fronds/ genotype), we expected that this target density would not represent a polygenic equilibrium but could serve as an approximate starting point to ensure that we did not overseed populations, which would impede visual genotype tracking.

**A3. Preliminary tests to explore the effects of nail polish dot colour and size on *L. minor* frond growth**

Before proceeding with the sustained range expansion experiment and using dots of various nail polish colours applied to *L. minor* frond surfaces to visually track the relative abundance of different genotypes, we tested the effects of nail polish dot colour and size on frond growth. For this trial, we used one genotype of *L. minor* (LM001 or G1) and one genotype of *Lemna x japonica* (LM004 or G4), previously collected from Vancouver, British Columbia and sterilized (Usui & Angert, 2024). We set up 3 x 6 cm-diameter plastic cups filled with 100 mL of N-medium (Appenroth et al., 1996). Two to four rafts were placed in a cup and dotted with a randomly selected nail polish colour from the Sally Hansen Insta-Dri line (a shade of red, orange, yellow, blue, purple, pink, black, and white). At the beginning of the trail, dots were applied on the centre of the fronds so that < 40% of their frond surface area was covered. We placed the cups in random order under grow lights at room temperature (~ 21°C). Some of the polish shades used in this trial differed from those in the main experiments since some were later replaced to increase visual contrast, but similar polishes from the same brand were used within each colour range. We applied an additional treatment factor for one genotype (LM001, *L. minor*) and colour treatment (black), varying dot size (dots covering >70% of the frond area). In total, there were 32 cups (8 colours x 3 replicates + 6 controls (non-dotted) + 2 large dot size replicates). We counted the total number of fronds in each replicate on days 0, 3, 5, 7, and 10.

To determine whether population growth was sensitive to dot size (i.e., > ~ 70% of frond area vs. ~ < 40% of frond area), we regressed the number of fronds (natural log-transformed) against day, dot size, and their interaction. This model was a simple linear regression, as the cup replicate did not explain any random variance. To determine if growth rates differed between nail polish colours applied to frond surfaces, we modelled the number of fronds (natural log-transformed) predicted by day, dot colour, and their interaction as a linear mixed-effect model with cup as a random effect, using the “lme4” R package (Bates et al., 2015) (n = 3, plus two control replicates without nail polish dots). We obtained estimated marginal means using the emmeans::lstmeans function (Lenth, 2022).

The first model suggested that both small and large dots depressed population growth over a short period, with small dots having a smaller effect than large dots (table S3). Therefore, to minimize the impact of nail polish application on growth, we only dotted fronds that would have less than ~ 40 % of their surface area covered; thereby, we ignored juvenile fronds until they reached a sufficient size, as qualitatively determined. Additionally, we found there were no significant differences between the nail polish colour applied and population growth over this short test period (figure S3, table S2).

**A4. Maintenance procedures for the sustained range expansion experiment**

Maintaining the duckweed populations in the sustained range expansion experiment involved refreshing the nutrient media, applying and re-applying dots to fronds, introducing additional *S. polyrhiza* propagules and mold mitigation. We refreshed the media and water levels every 2-4 days. Due to evaporation from the open lid, we first added fresh autoclaved-deionized water using a 1-10 mL Fisherbrand Elite Adjustable-Volume Pipette until the total media volume was returned to 100 mL. Some cups required greater water additions due to differences in incubator positions, but both treatments were equally affected due to grouping. We then removed 10 mL of media and replaced it with 10 mL of new 5% D-media to approximate chemostatic conditions. In earlier weeks, water and media were added and drawn by placing the pipette tip in large gaps between fronds at the water surface. In the latter weeks, when populations were denser, these tasks were achieved by placing the pipette tip against the cup walls. In both cases, when adding liquid, the pipette tip was held very close to the water surface and released slowly to minimize disturbance to fronds. Autoclave-sterilized pipette tips were replaced if they came into contact with cup contents before refreshing the water or media of the next cup in the series.

New *L. minor* fronds generated throughout the experiment required nail polish dots to be applied during attachment to the mother raft or shortly thereafter. Initially, we simply applied new dots to daughter fronds resting at the water surface, often supporting or slightly lifting the frond with a sterile bacteriological loop while dabbing the frond surface with nail polish at the end of a toothpick. However, due to unforeseen overcrowding midway through the experiment (likely due to overshooting equilibrium – figure S4), we later began lifting and redistributing fronds using sterile bacteriological loops to reduce clustering and prevent dispersion of the hydrophobic nail polish at the water surface when applying new dots. This technique quickly became impractical. In later weeks, we only dotted new fronds at the surface, representing a sample of the total frond composition. Additionally, throughout the experiment, but mostly in later weeks, we reapplied polish to fading dots, mainly red and pink as well as blue and purple; colours which looked similar once faded. Midway through the experiment, we also began re-dotting any fronds initially assigned to a dark green colour with white polish to increase visual contrast with black-dotted fronds. Doing so ensured that resident genotypes remained distinguishable in the following weeks.

We repeatedly replaced *S.* *polyrhiza* propagules to maintain competitive selection pressure in the exposed populations. The initial intent was to allow *S. polyrhiza* propagules to increase in abundance, thereby increasing selection pressure on the resident throughout the experiment. However, in many replicates, *S. polyrhiza* fronds began to die off, showing significant yellowing and necrosis by week six. *S. polyrhiza* produced many turions (dormant fronds) instead of vegetative fronds (Appenroth et al., 1996; Docauer, 1983; Jacobs, 1947). Turions likely do not exert the same competition and selection pressure due to their dormant physiology (Susplugas et al., 2000). Therefore, we supplemented exposed populations with new *S. polyrhiza* propagules in weeks 7, 9, and 12 to ensure that three healthy rafts were consistently present. A total of 9-44 fronds (an average of 27.3) and a total of 2 to 9 rafts (an average of 5.9 rafts) were added to exposed replicates over the course of the experiment in addition to the three initial *S. polyrhiza* rafts.

As significant mold accumulation appeared around week ten of the experiment, we undertook measures to slow its encroachment. For some cups, a substantial accumulation of mold on the water surface spreading over the fronds either yielded a patch of unhealthy and dying fronds or grew so thick that it obscured genotype markers and inhibited further tracking. To limit further spread, these replicates were removed from the incubator between weeks 10 and 14; their genotypic relative abundances were not recorded, and they were not used in the re-exposure experiment (5 populations: 2 unexposed, 3 exposed). The remaining replicates had varying levels of mold accumulation by the end of the experiment, but it either remained on cup walls or did not appear to deteriorate the health of fronds from visual assessments. In the cups where mold was present on cup walls, growing on dried, perished fronds, we gently wiped the inner walls and those of the other cup in its replicate pair with 0.5% Accel PREVention hydrogen peroxide wipes in an attempt to disinfect these areas.

Lastly, a handful of cups were accidentally disturbed at various points of the experiment. An exposed cup (cup 34) was bumped in week 4, causing substantial mixing to the duckweed community contained within. To mitigate this confounding factor within the replicate, we replaced this cup with another in close proximity that was originally intended for a different treatment, in which *S. polyrhiza* was also introduced to *L. minor.* The additional maintenance procedures for the third treatment never proceeded, so we considered this cup a suitable replacement for the one that had been bumped. An additional four cups (7, 8, 40, 49) were accidentally disturbed in mid-to-late weeks of the experiment, at which point no suitable replacements were available. We continued to maintain these cups and later used them for trait sampling (see appendix A7), but they were not included in the assessment of rapid evolution, where this confounding effect could have been more impactful.

**A5.** **Estimating final surface area occupied**

In addition to counting the number of fronds present in each cup at the end of the sustained exposure experiment, we estimated the total surface area occupied by duckweeds (unexposed: *L. minor*, exposed: *L. minor* + *S. polyrhiza*) for the paired replicates remaining in the final week (not overly contaminated with mold) (n = 34 cups). We used images from week 13 because the lighting conditions were more consistent in this image series. Prior to imaging, each cup was restored to a measured media level, ensuring that variations in media volume did not distort surface area measurements. To estimate surface area, we first used a customized macro script in ImageJ from Usui (2023) to transform these images to represent the intensity of green in each pixel. We then used the colour threshold tool to select the greenest areas of the image representing living tissue. We manually selected this threshold for each replicate based on the lighting conditions and identified the green areas using the reference (original) images. Using the cup's known diameter to set the image scale, we used the measure tool to calculate the area of the highlighted region.

**A6. Rapid evolution model outlier analysis**

We removed 12 extreme outliers from the rapid evolution model because we had reason to suspect they had arisen from genotype-tracking errors. In a few instances, between weeks 2-6, it appeared that one particular genotype was dotted with a nail polish colour corresponding to a different genotype in a given replicate, or vice versa. We attempted to fix these errors visually by re-dotting the fronds with the correct colour for the genotype, but may not have fixed or caught all of them. Unfortunately, detailed records about which cups these errors pertained to were not carefully documented. For this reason, we suspect that errors may have propagated in certain cup replicates across generations, resulting in extreme outliers. Nevertheless, we explored the composition of outliers and applied two additional model iterations of the linear model of rapid evolution (temporal change in relative abundance (∆RA) of *L. minor* genotypes predicted by an interaction between genotype and treatment with replicate number as a fixed effect). The first model only removed paired outliers (in the same cup population) that might have been swapped one-for-one. The second iteration only included outliers dotted with nail polish colours that were more likely to be mistaken for another colour (purple/blue, pink/red, white/black, see appendix A4). We report on these results in appendix B1.

**A7. Measuring *L. minor* trait plasticity in response to sustained range expansion exposure**

To measure morphological trait plasticity in *L. minor* between unexposed and exposed populations immediately following the sustained range expansion experiment, we haphazardly sampled eight rafts of each resident genotype from a subset of the unexposed (n = 4) and exposed (n = 5) populations. Five of these cups (two unexposed: 7, 49; and three exposed: cups 8, 34, 40) were accidentally physically disturbed more than other cups at some point during the experiment, leading to the potential for more mixing to occur (appendix A4). We selected these cups for trait sampling purposes because we believed that appropriate contrasts could still be made between treatments, with these cups being similarly disturbed. Additionally, sampling from these populations instead of the dwindling number of remaining cups not affected by substantial mold accumulation allowed for a larger sample size in the re-exposure experiment. We haphazardly sampled the remaining cups (cups: 20, 50, 51, 59) on the basis of containing combinations of genotype nail-polish colour assignments that minimized the risk of misidentifying genotypes. More specifically, despite reapplying nail polish throughout the experiment, purple and blue markers, as well as pink and red markers, were more difficult to distinguish from one another and posed a higher risk of misidentification or mismarking. This presented less of an issue in cups, where these colour combinations were used to track more visually distinct genotypes (e.g. one genotype from visually similar group 1: L-9495, L-9223, L-5576, L-5500, L-8623 and one genotype from group 2: L-8625, L-8784, L-7436). Still, for the selected cups, which we assessed to have a lower overall risk of genotype misidentification, we did not sample certain genotype-colour combinations (e.g., blue L-9495 and purple L-5500), resulting in an uneven distribution of sampling across replicates. This led to substantial variation in sample sizes for trait measurements.

For the sampled *L. minor* rafts, we first measured the length of the longest root on each raft using a standard ruler (±1 mm), from the thallus attachment to the root tip (Hess et al., 2022). For longer roots that did not hang straight when lifted from the water, we dragged the raft across dark paper, as in Jewell & Bell (2023a). This allowed the roots to lie approximately straight for measurement. Despite this technique, genotypes with longer roots likely had a greater margin of measurement error due to additional curvature. Next, we measured raft size, as the number of fronds containing any green (healthy) tissue, which may be indicative of short-distance dispersal capacity (e.g., fewer fronds per raft = higher dispersal due to earlier detachment from the stipule linking the parent and daughter fronds) (Usui & Angert, 2024) and has also been shown to be under selection in competitive environments (Usui & Angert 2026, Urquhart et al. 2026). We also measured frond area as the average area of two fronds per sampled raft (see Supplementary Information). Finally, we quantified a proxy index of root-to-shoot biomass allocation by dividing root length by our frond area (i.e., root length: frond area, mm/mm^2^). We assumed that fronds without visible roots were either juvenile or missing due to physical disturbance during population maintenance (i.e., from application of dots: see appendix A4, or from turbidity upon water and nutrient refreshment). Therefore, we removed samples with a root length of zero from the analysis (two samples).

**A8. Measuring *L. minor* functional traits to explore relationships with observed inter- and intraspecific selection**

We measured a set of functional traits for our eight *L. minor* genotypes to explore potential relationships with selection during the sustained range expansion experiment. In addition to our trait measurements on polygenic populations from the sustained range expansion experiment (appendix A7), we measured these same traits in *L. minor* in isogenic low- or high-nutrient populations. For each genotype, between August 9th and 15th, 2023, we seeded four 250 mL Erlenmeyer flasks with enough *L. minor* fronds to cover ~ 25% of the water surface area. We selected this starting value to maximize the number of replicates established with the *L. minor* stock on hand. These flasks contained either 100 mL of 5% D-media (isogenic, low-nutrients) or 100% D-media (isogenic, high-nutrients) (n = 2 per treatment). Fronds for the low-nutrient treatment were extracted from populations growing alongside the experimental populations in the sustained range expansion experiment in the incubator on 5% D-media since July 9th, 2023, while fronds for the high-nutrient treatment were extracted from stock populations grown on a heat mat at room temperature (~ 21 °C) and high-nutrient N-media for three months. We placed these populations in a random pattern on another shelf within the same incubator as the sustained range expansion experiment populations (same lighting conditions and temperature: 27 °C during the day, 25 °C at night, 16:8 hrs light-to-dark, under 30W LED Sublaster T5 fluorescent grow lights). Fresh nutrient medium was added periodically to restore the initial volume in each flask. The populations were left to grow for one month and reached equilibrium approximate densities ~2 weeks after seeding (visually estimated).

Between September 15th and 20th, 2023, we sampled ten rafts from each replicate population (N = 20) and measured root length, raft size, and frond area using the same procedures as in appendix S7. Separately, we measured isogenic, low-nutrient, per capita growth rates for each *L. minor* genotype (λ_L_) by seeding small plastic cups with 22 mL of 5% D-media with two rafts (n = 5 / genotype) and monitoring growth of new fronds over five days under high light conditions (under 30W LED Sublaster T5 fluorescent grow lights) on a 16:8 hour light-to-dark ratio, and at room temperature (~ 21 °C). Monitoring this growth at the experiment's temperature, 27°C, would have been ideal, but space limitations in the incubator prevented it.

**A9. Exploring functional trait variation between the *L. minor* genotypes**

To explore whether significant morphological trait variation existed between genotypes, to begin with, we examined variation in measured and derived traits (root length, raft size, frond area, and root length to frond area ratio) between genotypes within each treatment (polygenic-unexposed, polygenic-exposed, isogenic-low-nutrient, isogenic-high-nutrient) with linear regression models using the “stats” and “lme4” packages (Bates et al., 2015). Root length, frond area, and root length to frond area ratio were regressed with an interaction between *L. minor* genotype and treatment with cup replicate as a random variable. We natural-log transformed the response variables for the root length and frond area models, as this consistently improved adherence to model assumptions, as assessed using the “performance” and “DHARMa” packages (Hartig, 2022; Lüdecke et al., 2021). We similarly regressed raft size as a Poisson family model using the “lme4::glmer” function; however, the model fit was singular when the cup replicate was used as a random variable. Therefore, we treated cup replicate instead as a fixed effect. Because these data were significantly underdispersed—uncommon for count data—we used a quasi-Poisson family rather than a Poisson family, which better handles this scenario and yields smaller standard errors around the model estimates (Zeviani et al., 2014). We also investigated differences in isogenic low-nutrient growth rates among *L. minor* genotypes using simple linear regression with genotype as the predictor variable and λ_L_ as the response variable (n = 5). Finally, we repeated this modelling procedure, using hybrid status rather than genotype as a fixed effect, to explore whether these traits differed significantly between confirmed *L. minor* and *L. x japonica* genotypes.

**A10. Re-exposure experiment details**

We carefully transferred *L. minor* from all populations in the previous experiment, after trait sampling, to new cups containing fresh 100 mL of 5% D-media. We removed old propagules of *S. polyrhiza* and exposed all populations (previously exposed, n = 18; previously unexposed, n = 17) to three new propagules (rafts) of *S. polyrhiza* grown on 5% D-media for > 1 month. We placed all cups in the incubator for 12 days. However, the incubator broke on the morning of the 12th day and could no longer maintain a consistent temperature. All populations were promptly moved to a heat mat (Redi-Heat Heavy Duty Propagation Mat, Model RHD2105, 21x5", 150 W controlled with an Inkbird thermostat) set to a constant 27 °C under similar lighting conditions (Sunblaster T5 fluorescent lighting, at a similar height above the water surface).

**Appendix B. Supplementary Results**

**B1. Rapid evolution model outlier assessment & alternative models**

In assessing the composition of the 12 outliers in our main rapid evolution model, three of these outliers had a greater than average change in relative abundance over 14 weeks, and the other nine had lower than average changes (table S8). There are three pieces of evidence that point to these outliers being attributable to genotype misidentification rather than meaningful ecological variation. First, genotype L-8625 was the most common outlier, followed by L-8784. These two genotypes also had the smallest raft sizes (fewest fronds per raft) in low nutrient conditions. It is plausible that the tendency to release daughter fronds more quickly led to more instances where the genotype identity had to be assumed based on appearance and proximity to nearby frond identities after detaching from the mother raft, and therefore, more opportunities for mistaken identities to be propagated along a lineage. The second indicator is that white nail polish was the most common dot colour amongst these outliers. This colour treatment began as green dots in week 1, but we soon after applied green nail polish dots on top of the white as we discovered it was difficult to distinguish green from black nail polish dots. It is possible that applying extra nail polish dots to the surface led to reduced growth rates, which is supported by all four outliers with this colour having changes in smaller than average changes in relative abundance. Lastly, there were two cups with paired outlier points where one *L. minor* genotype showed an unexpectedly large increase in temporal relative abundance and the other an unexpectedly large decrease (cups 9 and 15). Furthermore, these paired outliers had nail polish colours that looked similar once faded, suggesting that these genotypes might have been mistaken for one another in certain replicates.

We found that an alternative rapid evolution model, only removing the four paired outliers that may have been swapped one-for-one resulted in an ANOVA p-value increase for the treatment by genotype interaction from 0.01429 to 0.088, but the L-9223 effect remained significant, p < 0.05 (table S9). In contrast, only excluding the seven outliers corresponding to nail polish colours that had nail polish dots reapplied during the experiment slightly decreased the ANOVA p-value of the treatment by genotype interaction (p = 0.0140) and the individual genotype selection estimates were minimally impacted.

**B2. Selection driven by intraspecific competition**

In addition to exposure-driven selection for particular genotypes by interspecific competition, intraspecific competition also contributed to changes in the relative abundance of *L. minor* genotypes (intraspecific selection) in both unexposed and exposed populations (figure 2B, figure S9). Intraspecific selection was strongest for the hybrid *Lemna x japonica* genotypes, which showed the greatest increase in relative abundance in both treatments (table S7). L-8784 increased by 6.17% [95% CI = 4.23, 7.65%] (p < 0.01) in the unexposed treatment. L-7436 also increased in relative abundance by 2.23% [95% CI = 0.29, 3.71%] (p < 0.01). Two of the four genotypes confirmed to be *L. minor* (L-8623, L-5576) showed significant decreases in relative abundance in unexposed populations. L-9223, whose identity has not been confirmed, decreased the most in frond area due to intraspecific selection (-4.18%, [95% CI = -6.12, -2.70%], p < 0.01).

Some functional traits were significantly correlated with selection driven by intraspecific competition (figure S9, table S14). Frond area on high nutrients was the strongest predictor (β = 1.6 % / mm^2^, p = 0.0025, followed by frond area plasticity (β = 0.19 %, p = 0.0029), and root length on high nutrients (β = 0.15 % / mm, p = 0.014), which suggests that *Lemna* genotypes with larger fronds initially, greater frond area plasticity, and longer roots were favoured by intraspecific competition. This corresponds to the selection for the hybrid genotypes L-8784 and L-7436 in unexposed populations (figure 2B).

**Additional references**

Susplugas, S., Srivastava, A., & Strasser, R. J. (2000). Changes in the photosynthetic activities during several stages of vegetative growth of *Spirodela polyrhiza*: Effect of chromate. Journal of Plant Physiology, 157(5), 503–512.

Urquhart, C. A., Usui, T., Angert, A. L., & Williams, J. L. (2026). Eco-evolutionary dynamics are shaped by competition in experimental range expansions. *bioRxiv,* 2026-06.

Usui, T., Angert, A. L. (2026). Competition enables rapid adaptation to a warming range edge in a model plant community. *Science, 392*, eads4664.

**Appendix C. Supplementary Figures**

**Figure S1**. Finding isogenic equilibrium density points for eight *Lemna minor* genotypes. The trend line represents generalized additive (GAM) models with a k = 4 smoothing parameter. The grey zone indicates 95% confidence intervals from the trend line. Boxes are the interquartile range, whiskers are the full range, and horizontal lines are medians of population growth rate (surface area spread, cm^2^ per day) values across four replicates at each sampling date.

**
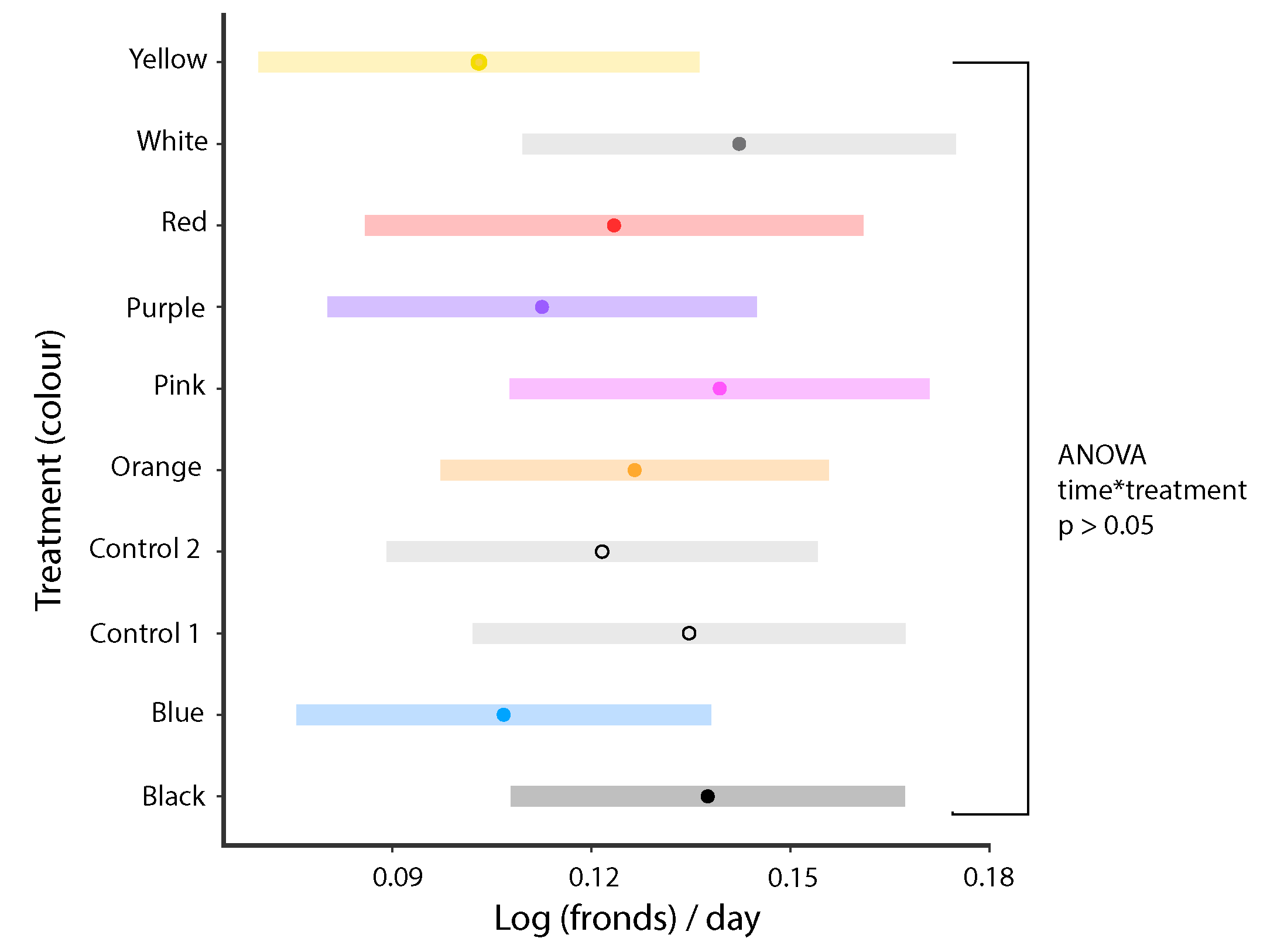
Figure S2.** Impacts of applying small dots (< ~ 40% area) of various shades of Sally Hansen Insta-Dri nail polish on population growth (frond production) in the *Lemna minor* complex over 10 days (n = 3 replicates per treatment). The x-axis indicates the slope of linear regressions (change in fronds produced per day) produced by the following mixed effects model: Ln(number of fronds) = time*treatment + (1 | replicate). “Control 1” and “Control 2” treatments refer to replicate populations where fronds were not dotted with nail polish (grey bars and open circles). The growth trials included two genotypes (one *Lemna minor* and one *Lemna x japonica* hybrid) collected from the Greater Vancouver Area, British Columbia, Canada. Dots indicate estimated marginal mean slope values, and bars represent 95% confidence intervals.

**Figure S3.** Influential outliers identified by Cook's distance for differences in *Lemna minor* genotype relative abundances over 14 weeks in unexposed populations and populations exposed to *Spirodela polyrhiza*. The y-axis indicates Cook's distance values of each observation (index). Outliers above the threshold of 7 / N, where N = 328 observations. (N = 328, n_unexposed_ = 168, n_exposed_ = 168).

**
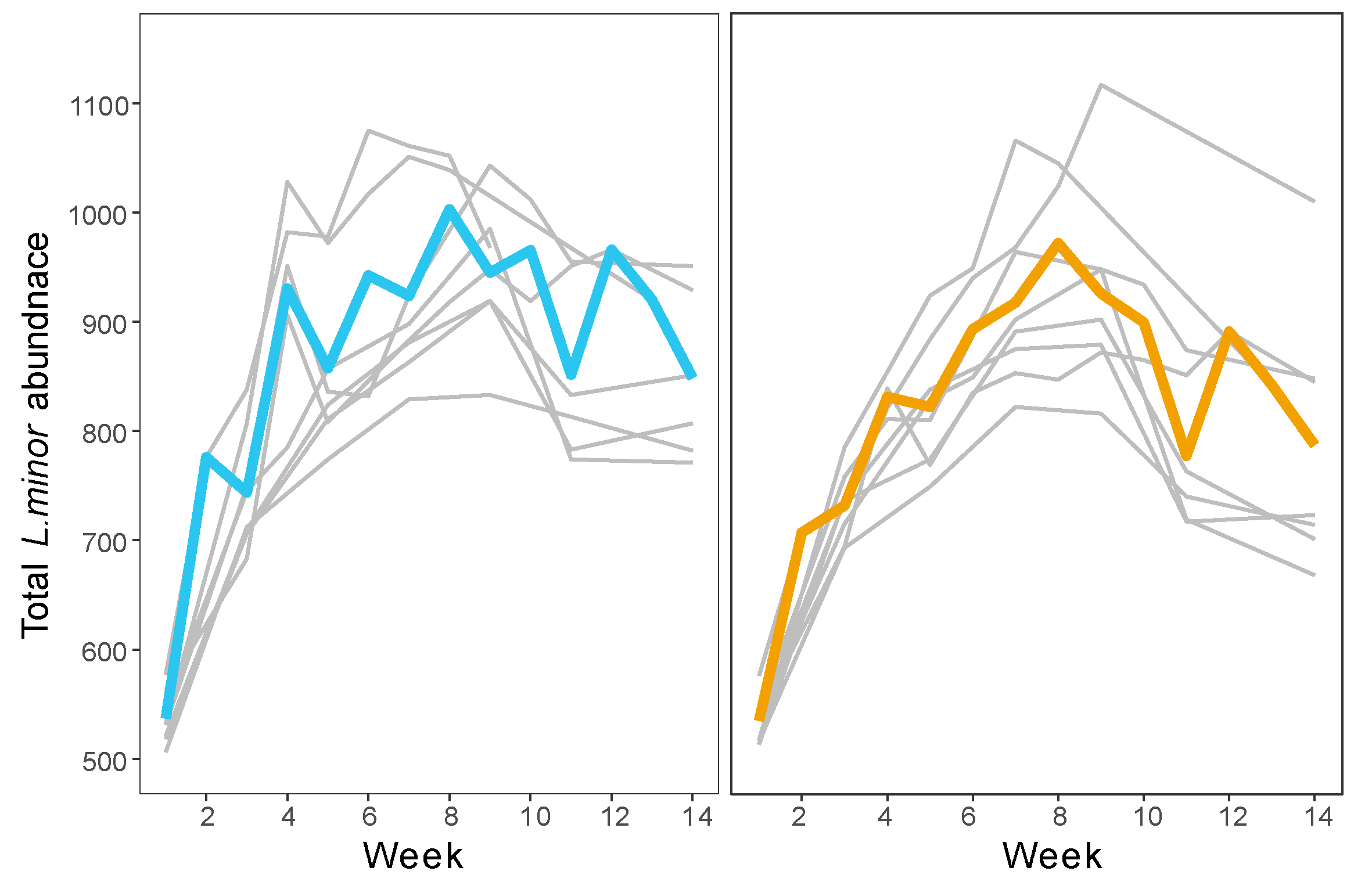
**

**Figure S4**. Total abundance of *Lemna minor* in the first eight replicate populations composed of eight *L. minor* genotypes where half of the populations were exposed to *Spirodela polyrhiza* at low density (right) and the other half were left unexposed (left). The means across these replicates are plotted with thick lines (blue = unexposed populations, orange = exposed populations).

**
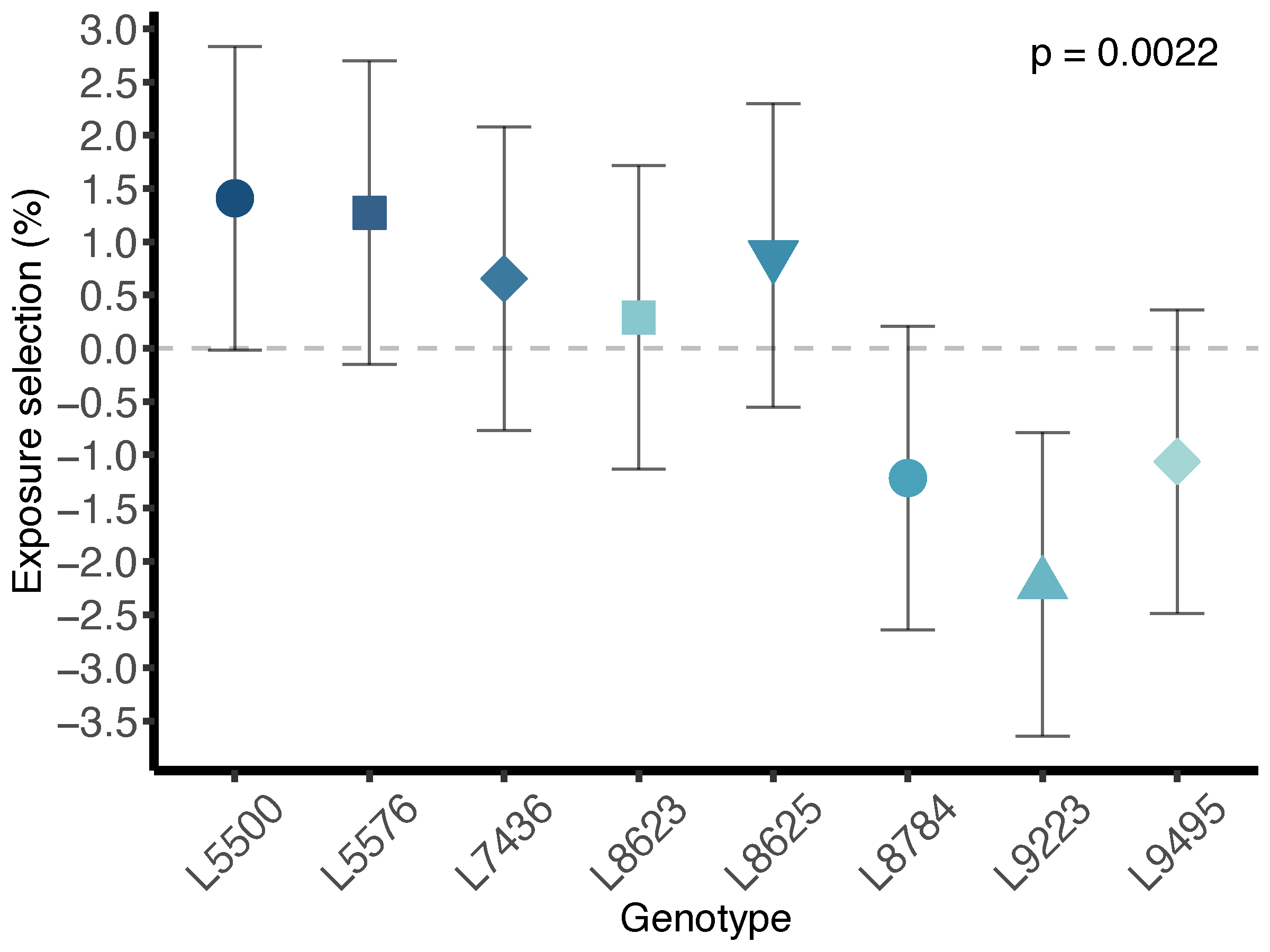
**

**Figure S5.** Sensitivity analysis of rapid evolution models for results generated with a) a 1% genotype misidentification rate and b) a 10% genotype misidentification rate averaged across 999 bootstrap replicates. *Lemna minor* genotype identities were randomly swapped within cup replicates and within visual similarity groups (group 1: L-5500, L-5576, L-8623, L-9495; group 2: L-8625, L-8784, L-7436). The genotype L-9223 was not included in these random misidentifications as it was visually distinct. Exposure-driven selection (+/- 95% Tukey-adjusted confidence intervals) (y-axis) was calculated as the difference in estimated marginal means of the change in relative abundance over 14 weeks (∆RA = RA_week14_ - RA_week0_ (%)) between treatments, where populations were either exposed to low densities of a competitor duckweed, *Spirodela polyrhiza* (n = 20), or unexposed (n = 21) (exposure-driven selection % = ∆RA_Exposed_ - ∆RA_Unexposed_). (N = 328, n_unexposed_ = 168, n_exposed_ = 168).


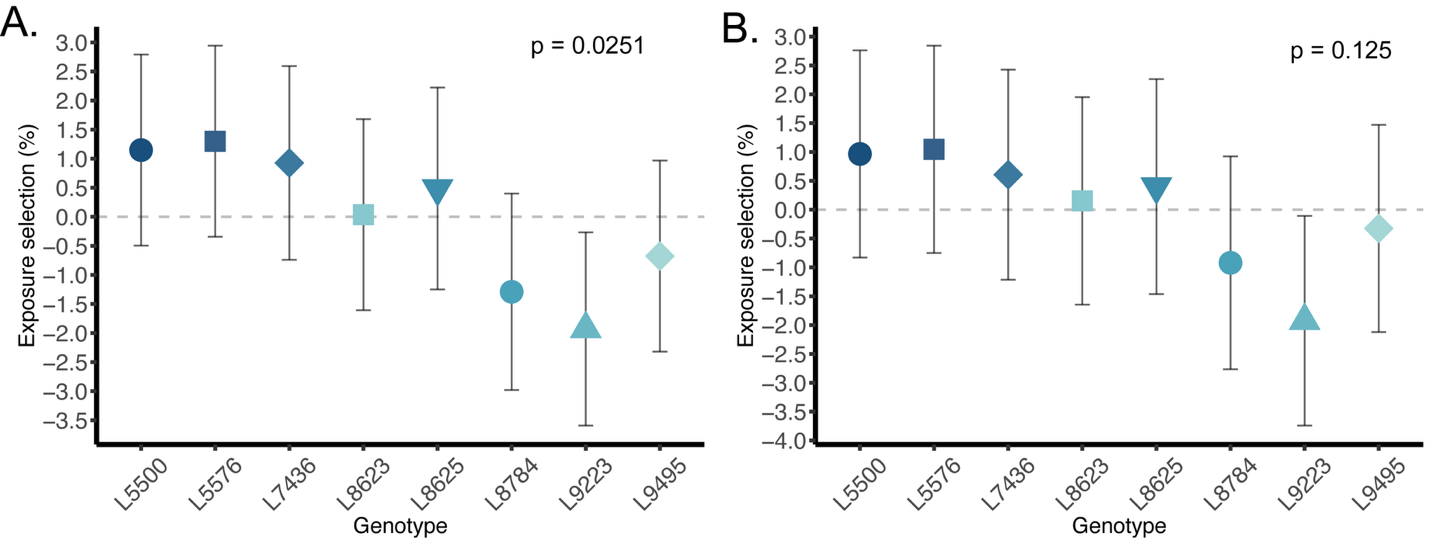


**Figure S6.** Alternative rapid evolution model. Predicted exposure-driven selection on eight resident *Lemna minor* genotypes (+/- 95% Tukey-adjusted confidence intervals). Exposure-driven selection was calculated as the difference in the change in relative abundance over 14 weeks (∆RA = RA_week14_ - RA_week0_ (%)) between paired replicate populations either exposed to low densities of a competitor duckweed, *Spirodela polyrhiza,* or unexposed (exposure-driven selection % = ∆RA_Exposed_ - ∆RA_Unexposed_). Positive values indicate selection for and negative values selection against particular genotypes. The ANOVA p-value is in the upper-right corner. (N = 136, n_replicates_ = 17).


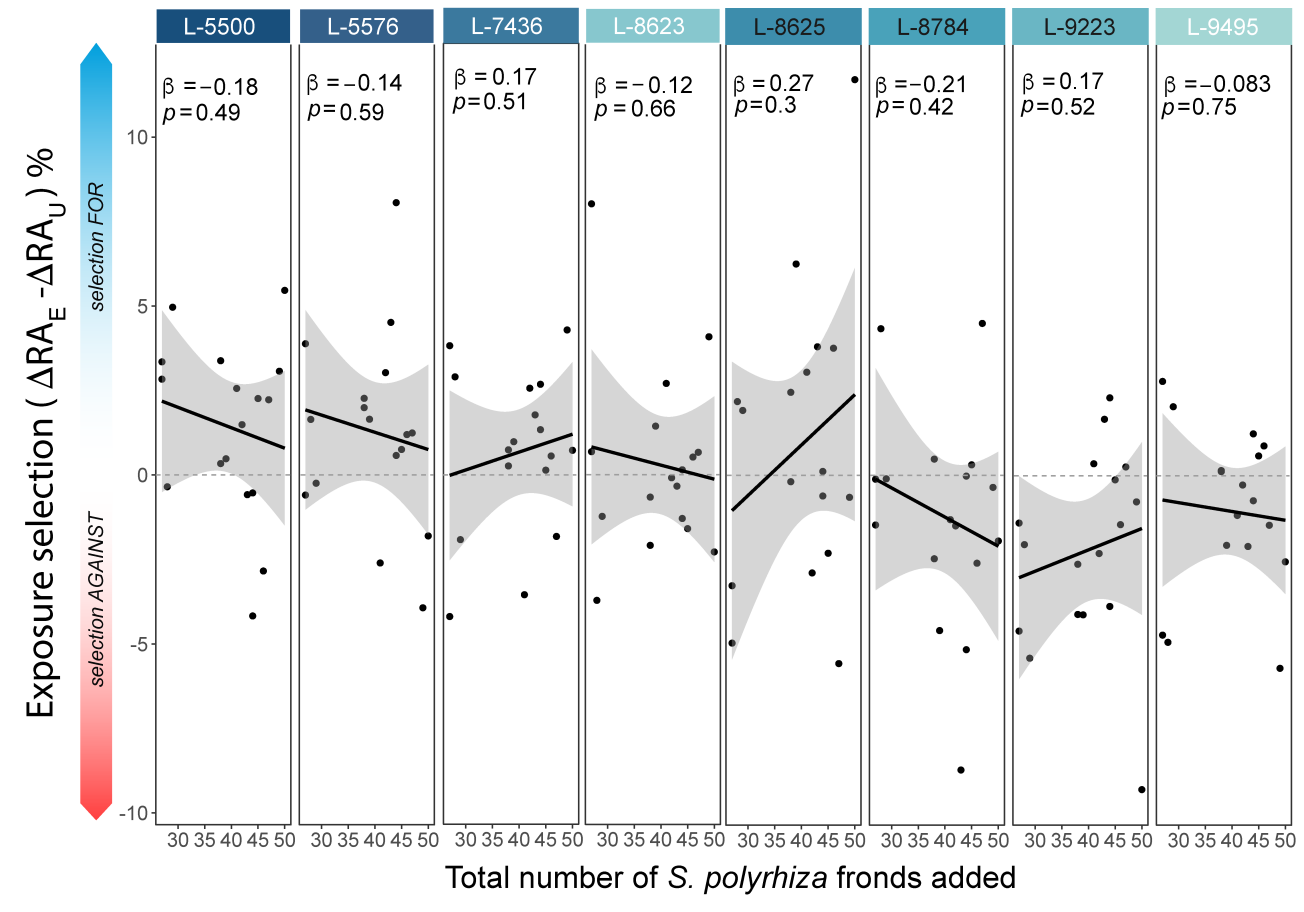


**Figure S7.** Observed range-shifter exposure-driven selection (%) experienced for eight *Lemna minor* genotypes, with the total number of range-shifter propagules (*Spirodela polyrhiza* fronds) added to each replicate. The y-axis is the difference in observed ΔRA (changes in relative abundance for *L. minor* resident genotypes over 14 weeks) between populations within the same replicate, either exposed to (E) and unexposed to (U) *S. polyrhiza* at low density (Exposure-driven selection % = ΔRA_E_ - ΔRA_U_). Positive values indicate directional exposure-driven selection “for” (blue), and negative values indicate directional selection “against” (red). The x-axis is the total number of *S. polyrhiza* fronds added to exposed populations during a 14-week-long sustained range expansion experiment, including the initial number of fronds and the additional fronds added at weeks 7, 9, and 12. The trendlines are simple linear regressions (β = regression coefficient) bounded by 95% confidence interval bands (grey bands). (N = 136, n_replicates_ = 17).


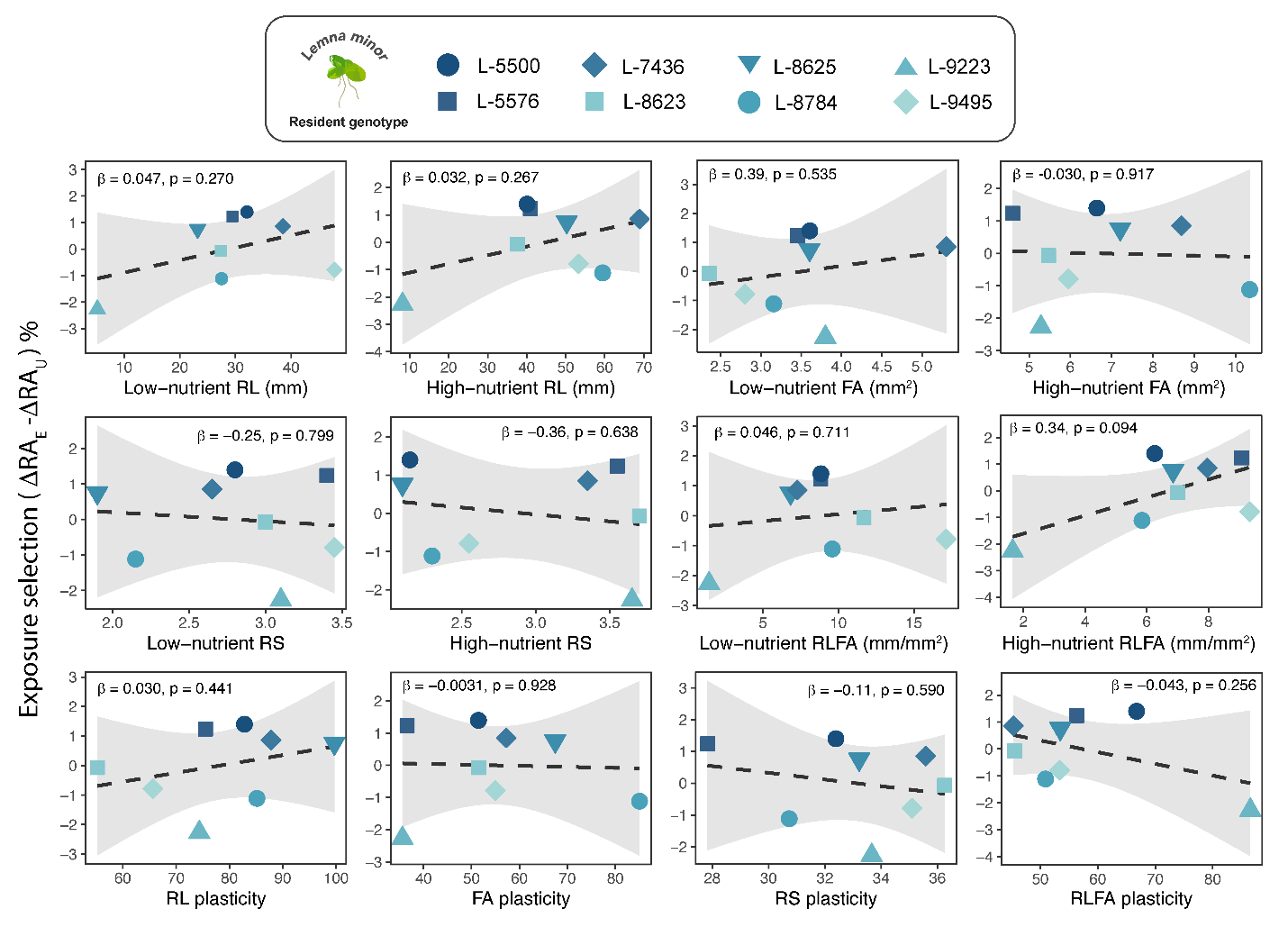


**Figure S8**. Exploratory univariate linear regressions (black lines, β = regression coefficient) of model-derived exposure-driven selection (%) experienced by eight *Lemna minor* (resident) genotypes in polygenic cultures with mean trait values of sampled rafts across various nutrient and competitive environments. The y-axis is the difference in ΔRA (changes in relative abundance of over 14 weeks) between *L. minor* populations exposed to (E) or unexposed (U) to *Spirodela polyrhiza* at low density (exposure-driven selection % = ΔRA_E_- ΔRA_U_). “High nutrient” and “Low nutrient” indicate that trait means are derived from isogenic populations growing on high nutrient media or low nutrient media, respectively. λ = per capita low density growth rate, RL = longest root length of raft (mm), FA = average frond area (mm^2^), RS = raft size (number of fronds alive), RLFA = root length to frond area ratio (mm/mm^2^). Trait plasticity was calculated as the coefficient of variation in traits across *L. minor* rafts sampled from isogenic populations grown under high or low nutrient conditions, as well as polygenic populations exposed or unexposed to *Spirodela polyrhiza* at low density. The grey bands are 95% confidence intervals.


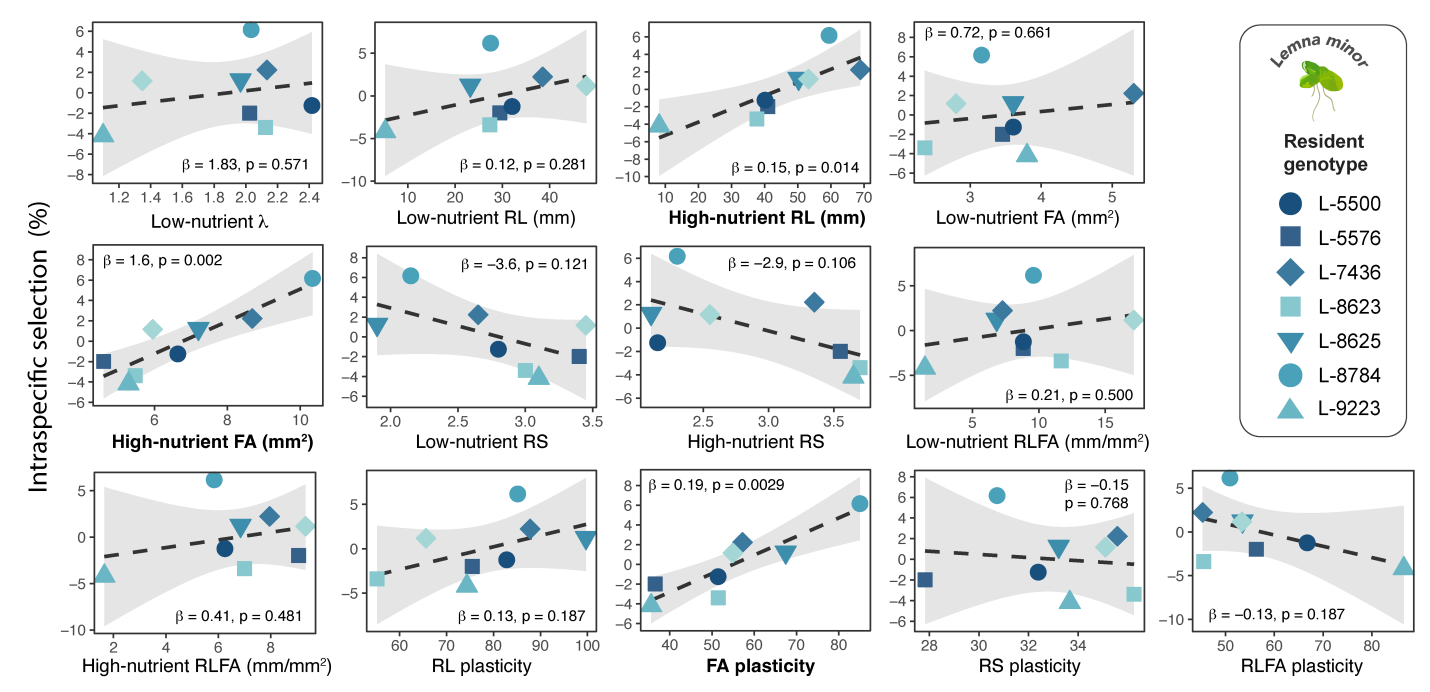


**Figure S9.** Exploratory univariate linear regressions (black lines, β = regression coefficient) of model-derived intraspecific competition-driven selection (%) experienced by eight *Lemna minor* (resident) genotypes in polygenic cultures with mean trait values of sampled rafts across various nutrient and competitive environments. The y-axis is the change in relative abundance over 14 weeks in *L. minor* populations (intraspecific-driven selection %, ΔRA_U_). “High nutrient” and “Low nutrient” indicate that trait means are derived from isogenic populations growing on high nutrient media or low nutrient media, respectively. λ = per capita low density growth rate, RL = longest root length of raft (mm), FA = average frond area (mm^2^), RS = raft size (number of fronds alive), RLFA = root length to frond area ratio (mm/mm^2^). Trait plasticity was calculated as the coefficient of variation in traits across *L. minor* rafts sampled from isogenic populations grown under high or low nutrient conditions, as well as polygenic populations exposed or unexposed to *Spirodela polyrhiza* at low density. The grey bands are 95% confidence intervals. Bolded axis titles indicate significant regressions (p < 0.05).

**Appendix D. Supplementary Tables**

**Table S1.** Identities and collection locations of eight duckweed genotypes identified as *Lemna minor* by outdated genetic markers selected from the Rutgers Duckweed Stock Cooperative (RDSC). “Genotype” is the name we refer to each accession, whereas “Landolt ID” is the ID assigned by the Landolt collection. “Hybrid?” indicates which genotypes have recently been identified as hybrids between *Lemna minor* and *Lemna japonica* (Y = yes, N = no, ? = has not yet been tested with updated genetic markers). Coordinates for each locality were georeferenced using Google Maps. ND = distance from the genotype accession location to the nearest occurrence of *Spirodela polyrhiza* (km). Coordinates for each locality were georeferenced using Google Maps.

| Genotype | RDSC Serial # | Landolt ID | Hybrid? | Country | Locality | Lat. (°) | Long. (°) | ND (km) |
| --- | --- | --- | --- | --- | --- | --- | --- | --- |
| L-5500 | 1007 | 5500 | N | Ireland | Blarney, County Cork | 51.9 | -8.6 | 33.4 |
| L-5576 | 370 | 9223 | N | United Kingdom | Wales, Machynlleth | 52.6 | -3.9 | 40.8 |
| L-8623 | 35 | 8623 | N | Denmark | Ijland Alborg | 57.0 | 9.9 | 13.9 |
| L-8625 | 157 | 8625 | Y | Norway | Oslo-Honefoss | 60.2 | 10.3 | 24.9 |
| L-8784 | 330 | 8784b | Y | Sweden | Simrishamn | 55.6 | 14.3 | 10.9 |
| L-9223 | 687 | - | ? | United Kingdom | Wales | 52.1 | -3.8 | 17.8 |
| L-9495 | 387 | 9495 | N | Norway | Stavanger | 59.0 | 5.7 | 15.2 |
| L-7436 | 366 | 7436 | Y | Russia | Onega, Kondopogskaja Guba | 63.9 | 38.1 | 502.4 |

**Table S2.** ANOVA summary of a mixed linear regression of the natural log of *Lemna minor* fronds present in a cup predicted by an interaction between day since the beginning of the experiment and colour of Sally Hansen Insta-Dri nail polish applied in small dots (< ~ 40% area) to frond surfaces (n = 3 replicates per treatment). The effect of colour also included two undotted (control) treatments. Genotype (one *Lemna minor* and one *Lemna x japonica* hybrid) and replicate were treated as fixed and random effects, respectively. p < 0.005 = ***, p < 0.05 = *. (N= 173).

|  | Sum Sq. | Mean Sq. | NumDf | DenDf | F-value | P-value |  |
| --- | --- | --- | --- | --- | --- | --- | --- |
| Day | 39.079 | 39.08 | 1 | 121.89 | 1396.23 | **< 2.2e^-16^** |  |
| Colour | 0.686 | 0.076 | 9 | 129.32 | 2.72 | **0.006129** |  |
| Genotype | 0.909 | 0.91 | 1 | 30.68 | 32.48 | **2.99e^-16^** |  |
| Day:colour | 0.377 | 0.042 | 9 | 126.90 | 1.50 | 0.1560 |  |
|  |  |  |  |  |  |  |  |

**Table S3.** Impacts of applying dots of one colour of Sally Hansen Insta-Dri nail polish (garnet) of varying sizes to *Lemna minor* fronds on population growth (frond production) over 10 days. Large dots (L) encompassed ~ 70 % of frond surface area, whereas small dots (S) were applied to < ~ 40 % frond area (n = 5). Fronds were left undotted in the control treatment (n = 10 replicates). Growth rate is the slope of regressions produced by the following linear model: Ln(Number of fronds) = time*treatment; indicative of the change in fronds produced per day. This growth trial included a single *L. minor* genotype collected from the Greater Vancouver Area, British Columbia, Canada. SE = standard error.

| Contrast | Estimated Δ growth rate | SE | df | t.ratio | p-value |
| --- | --- | --- | --- | --- | --- |
| Control - L | 5.26 | 0.63 | 34 | 8.40 | < 0.0001 |
| Control - S | 3.22 | 0.69 | 34 | 4.67 | 0.0001 |
| L - S | -2.04 | 0.67 | 34 | -3.07 | 0.0113 |

**Table S4.** Assessment of mold presence in week 14 of the remaining populations in the sustained range expansion experiment. All = all populations, U = unexposed *Lemna minor* populations, E = *L. minor* populations exposed to *Spirodela polyrhiza.* “Present” is the number of populations with visible patches of mold and the percentage out of all replicates remaining (all: N = 41, n_unexposed_ = 21, n_exposed_ = 20). “Only on walls” indicates when mold was present but not visibly growing over duckweed. “Large patches” were noted when mold was overgrowing > 2 duckweed rafts. The remaining columns denote the instances of mold of different colours. The percentages listed for columns 3 to 8 are relative to the number of populations where mold was present.

| Treatment | Present | Only on walls | Large patch | black | black/grey | grey | white |
| --- | --- | --- | --- | --- | --- | --- | --- |
| All | 19 (46%) | 8 (42%) | 9 (47%) | 10 (53%) | 2 (11%) | 4 (21%) | 3 (16%) |
| U | 9 (43%) | 5 (55%) | 4 (44%) | 5 (55%) | 1 (11%) | 3 (33%) | 0 (0%) |
| E | 10 (50%) | 3 (30%) | 5 (50%) | 5 (50%) | 1 (10%) | 1 (10%) | 3 (30%) |

**Table S5.** ANOVA summary of a linear regression between temporal change in the relative abundance of eight *Lemna minor* duckweed genotypes and a treatment by genotype interaction with replicate number as a fixed effect (ΔRA = treatment*genotype + replicate), where treatment has two levels, unexposed or exposed to the duckweed *Spirodela polyrhiza* at low density. p < 0.005 = ***, p < 0.05 = *. (N _total_ = 316, n_Exposed_ = 154, n_Unexposed_ = 162).

|  | Df | Sum Sq. | Mean Sq. | F-value | p-value |  |
| --- | --- | --- | --- | --- | --- | --- |
| Treatment | 1 | 0.1 | 0.09 | 0.0144 | 0.9047 |  |
| Genotype | 7 | 3292.0 | 470.29 | 73.1859 | < 2e-16 | *** |
| Replicate | 23 | 38.7 | 1.68 | 0.2616 | 0.99989 |  |
| Treatment:genotype | 7 | 115.2 | 16.46 | 2.5614 | 0.01429 | * |
| Residuals | 277 | 1780.0 | 6.43 |  |  |  |

**Table S6.** Estimated marginal means of linear regression slopes between temporal change in the relative abundance of eight *Lemna minor* duckweed genotypes (ΔRA) and a treatment by genotype interaction with replicate number as a fixed effect (ΔRA = treatment*genotype + replicate), where treatment has two levels, unexposed (U) or exposed (E) to the duckweed *Spirodela polyrhiza* at low density. SE = standard error, CI = confidence interval. P-values were adjusted using the Bonferroni and Tukey methods with the “emmeans” R package, but 95% CI were presented only with the Bonferroni method because they were very similar across the adjustment methods. (N _total_ = 316, n_Exposed_ = 154, n_Unexposed_ = 162). Estimates were scaled to add to 0 for each treatment, and the 95% CIs were also scaled accordingly.

| Treatment | Genotype | ΔRA Estimate (%) | SE | P-value (Bonferroni) | 95% CI (Bonferroni) | P-value (Tukey) |
| --- | --- | --- | --- | --- | --- | --- |
| E | L-5500 | 0.15 | 0.55 | 1.00 | [-1.79, 1.63] | 1.00 |
| U | L-5500 | -1.25 | 0.55 | 0.39 | [-3.19, 0.23] | 0.33 |
| E | L-5576 | -0.75 | 0.55 | 1.00 | [-2.69, 0.73] | 0.95 |
| U | L-5576 | -1.99 | 0.54 | <0.01 | [-3.88, -0.55] | <0.01 |
| E | L-8623 | -3.46 | 0.55 | <0.01 | [-5.40, -1.98] | <0.01 |
| U | L-8623 | -3.39 | 0.54 | <0.01 | [-5.29, -1.96] | <0.01 |
| E | L-8625 | 2.00 | 0.60 | 0.02 | [-0.09, 3.64] | 0.02 |
| U | L-8625 | 1.25 | 0.57 | 0.45 | [-0.74, 2.78] | 0.37 |
| E | L-8784 | 5.04 | 0.58 | <0.01 | [3.01, 6.63] | <0.01 |
| U | L-8784 | 6.17 | 0.55 | <0.01 | [4.23, 7.65] | <0.01 |
| E | L-9223 | -6.45 | 0.57 | <0.01 | [-8.44, -4.92] | <0.01 |
| U | L-9223 | -4.18 | 0.55 | <0.01 | [-6.12, -2.70] | <0.01 |
| E | L-9495 | 0.38 | 0.55 | 1.00 | [-1.56, 1.86] | 1.00 |
| U | L-9495 | 1.17 | 0.54 | 0.51 | [-0.73, 2.60] | 0.40 |
| E | L-7436 | 3.08 | 0.55 | <0.01 | [1.14, 4.57] | <0.01 |
| U | L-7436 | 2.23 | 0.55 | <0.01 | [0.29, 3.71] | <0.01 |

**Table S7.** Pairwise post-hoc contrasts between temporal changes in the relative abundance of eight *Lemna minor* genotypes over 14 weeks between populations exposed to (E) or unexposed to (U) *Spirodela polyrhiza* at low density (i.e. exposure selection % = ΔRA_E_ - ΔRA_U_. Contrasts were performed using the emmeans “contrast” function, with family-wise p-values adjusted using the “mvt” method. Exposure selection estimates were scaled to add to 0. (N _total_ = 316, n_Exposed_ = 154, n_Unexposed_ = 162).

| Genotype | Exposure selection  (ΔRA_E_ - ΔRA_U_) (%) | SE | Df | T-ratio | P-value |
| --- | --- | --- | --- | --- | --- |
| L-5500 | 1.40 | 0.81 | 277 | 1.72 | 0.0865 |
| L-5576 | 1.24 | 0.80 | 277 | 1.54 | 0.1260 |
| L-8623 | -0.070 | 0.80 | 277 | -0.091 | 0.9277 |
| L-8625 | 0.75 | 0.86 | 277 | 0.87 | 0.3827 |
| L-8784 | -1.12 | 0.84 | 277 | -1.34 | 0.1819 |
| L-9223 | -2.27 | 0.82 | 277 | -2.77 | **0.0060** |
| L-9495 | -0.79 | 0.80 | 277 | -0.98 | 0.3264 |
| L-7436 | 0.85 | 0.81 | 277 | 1.05 | 0.2965 |

**Table S8.** Outliers identified by a Cook’s distance threshold of 7/sample size for the model predicting temporal change in the relative abundance of eight *Lemna minor* duckweed genotypes over 14 weeks (ΔRA) with a treatment-by-genotype interaction and replicate number as a fixed effect (ΔRA = treatment*genotype + replicate). Treatment has two levels: unexposed (U) or exposed (E) to the duckweed *Spirodela polyrhiza* at low density. “Cup ID” corresponds to separate population cups with two per replicate block. “Nail polish colour” corresponds to the colour of nail polish applied in small dots to the surface of *L. minor* fronds. “Anomaly” is the deviation in ΔRA from the average for that genotype and treatment.

| **Cup ID** | **Replicate** | ***L. minor* genotype** | **Nail polish colour** | **Treatment** | Δ**RA** | **Anomaly (**Δ**RA -** Δ**RA_avg._)** |
| --- | --- | --- | --- | --- | --- | --- |
| 5 | 2 | L8784 | white | E | 14.45 | -9.33 |
| 26 | 9 | L9223 | white | E | 3.70 | -9.77 |
| 26 | 9 | L8784 | black | E | -1.06 | 6.18 |
| 28 | 10 | L8784 | white | U | 16.13 | -9.68 |
| 34 | 12 | L8625 | black | E | 10.86 | -8.49 |
| 39 | 15 | L9223 | red | U | -10.33 | 5.63 |
| 39 | 15 | L5500 | purple | U | 5.34 | -6.50 |
| 42 | 16 | L8625 | pink | E | -5.83 | 8.20 |
| 45 | 18 | L7436 | orange | U | 11.21 | -8.75 |
| 52 | 21 | L8625 | orange | E | 10.84 | -8.47 |
| 53 | 22 | L8625 | white | U | 10.21 | -8.35 |
| 59 | 25 | L8625 | yellow | U | 7.67 | -5.81 |

**Table S9.** a) ANOVA summary and b) pairwise post-hoc contrasts of two alternative models of rapid evolution in *Lemna minor* upon sustained exposure to *Spirodela polyrhiza* over 14 weeks, varying by the outliers excluded. Model 1 excludes four paired outliers (two per cup) which may have been mistaken for each other in their respective replicates (N = 324). Model 2 excludes seven outliers that were dotted with nail polish colours that tended to be reapplied to fronds more than other colours, potentially inhibiting growth rates (N = 321). Both models were calibrated as simple linear regressions between temporal change in the relative abundance of eight *Lemna minor* duckweed genotypes and a treatment-by-genotype interaction with replicate number as a fixed effect (ΔRA = treatment*genotype + replicate), where treatment has two levels, unexposed or exposed to the duckweed *S. polyrhiza* at low density. Contrasts were performed using the emmeans “contrast” function, with family-wise p-values adjusted using the “tukey” method. p < 0.005 = ***, p < 0.05 = *. (N= 320).

|  |  | **Df** | **Sum Sq.** | **Mean Sq.** | **F-value** | **p-value** |  |
| --- | --- | --- | --- | --- | --- | --- | --- |
| Model 1 | Treatment | 1 | 0.2 | 0.18 | 0.021 | 0.885 |  |
|  | Genotype | 7 | 3749.4 | 535.63 | 63.25 | **<2.2e^-16^** | *** |
|  | Replicate | 23 | 1.0 | 0.04 | 0.005 | 1.000 |  |
|  | Treatment:genotype | 7 | 106.4 | 15.20 | 1.79 | 0.0881 | . |
|  | Residuals | 285 | 2413.3 | 8.47 |  |  |  |
| Model 2 | Treatment | 1 | 0.2 | 0.21 | 0.029 | 0.865 |  |
|  | Genotype | 7 | 3411.3 | 487.33 | 66.06 | **<2.2e^-16^** | *** |
|  | Replicate | 23 | 36.0 | 1.56 | 0.21 | 0.999 |  |
|  | Treatment:genotype | 7 | 132.6 | 18.94 | 2.57 | **0.0140** | * |
|  | Residuals | 282 | 2080.5 | 7.38 |  |  |  |
|  |  |  |  |  |  |  |  |
| Model 1 | **Genotype** | **Estimate** | **SE** | **DF** | **t.ratio** | **p.value** |  |
|  | L5500 | 1.50 | 0.93 | 285 | 1.62 | 0.1073 |  |
|  | L5576 | 1.33 | 0.92 | 285 | 1.44 | 0.1513 |  |
|  | L7436 | 0.49 | 0.92 | 285 | 0.53 | 0.5965 |  |
|  | L8623 | 0.02 | 0.92 | 285 | 0.02 | 0.9826 |  |
|  | L8625 | 0.53 | 0.92 | 285 | 0.58 | 0.5635 |  |
|  | L8784 | -1.01 | 0.93 | 285 | -1.08 | 0.2805 |  |
|  | L9223 | -2.17 | 0.94 | 285 | -2.30 | **0.0220** | * |
|  | L9495 | -0.70 | 0.92 | 285 | -0.76 | 0.4504 |  |
| Model 2 | L5500 | 1.49 | 0.87 | 282 | 1.72 | 0.0866 | . |
|  | L5576 | 1.32 | 0.86 | 282 | 1.54 | 0.1247 |  |
|  | L7436 | 0.49 | 0.86 | 282 | 0.57 | 0.5716 |  |
|  | L8623 | 0.02 | 0.86 | 282 | 0.02 | 0.9825 |  |
|  | L8625 | 1.47 | 0.88 | 282 | 1.67 | 0.0957 | . |
|  | L8784 | -1.31 | 0.88 | 282 | -1.48 | 0.1401 |  |
|  | L9223 | -2.22 | 0.88 | 282 | -2.53 | **0.0121** | * |
|  | L9495 | -0.70 | 0.86 | 282 | -0.81 | 0.4179 |  |
|  |  |  |  |  |  |  |  |
|  |  |  |  |  |  |  |  |

**Table S10.** ANOVA results of morphological plasticity linear models where measured traits are predicted by an interaction between treatment (either unexposed “U” or exposed to “E” *Spirodela polyrhiza* at low density for 14 weeks) and *Lemna minor* genotype. The models in (A) were constructed as linear mixed models with cup replicate ID as a random factor (root length: N = 447, 22-40 per treatment and genotype; frond area: N = 478, 21-40; root length/ frond area: N = 470, 21-40). Model (B) is a quasi-Poisson family generalized linear model (N = 490, 24-40 per treatment and genotype). Bolded values indicate p < 0.05. Resid. = residual. Df = degrees of freedom. Num = numerator. Den = denominator.

| **A.** |  |  |  |  |  |  |  |
| --- | --- | --- | --- | --- | --- | --- | --- |
|  |  | **Sum Sq** | **Mean Sq** | **NumDf** | **DenDf** | **F-Value** | **P-Value** |
| Root length (mm) | Treatment | 23.8 | 23.85 | 1 | 6.87 | 1.12 | 0.326 |
|  | Genotype | 4262.0 | 608.86 | 7 | 426.82 | 28.59 | **< 2.2e^-16^** |
|  | Treatment:genotype | 680.0 | 97.15 | 7 | 426.82 | 4.56 | **6.298e^-05^** |
|  |  |  |  |  |  |  |  |
| Frond area (mm^2^) | Treatment | 0.00 | 0.0004 | 1 | 6.61 | 0.0018 | 0.967 |
|  | Genotype | 38.36 | 5.48 | 7 | 458.68 | 26.937 | **< 2.2e^-16^** |
|  | Treatment:genotype | 5.95 | 0.85 | 7 | 458.68 | 4.18 | **0.000177** |
|  |  |  |  |  |  |  |  |
| Root length: frond area (mm/mm^2^) | Treatment | 3.10 | 3.10 | 1 | 7.28 | 0.508 | 0.498 |
|  | Genotype | 1096.77 | 156.82 | 7 | 448.19 | 25.69 | **< 2.2e^-16^** |
|  | Treatment:genotype | 164.78 | 23.54 | 7 | 448.19 | 3.86 | **0.000435** |
| **B.** |  |  |  |  |  |  |  |
|  |  | **Df** | **Deviance** | **Resid. Df** | **Resid. Dev.** | **F-value** | **P-value** |
| Raft size (# fronds per raft) | Treatment | 1 | 0.37 | 488 | 163.51 | 1.34 | 0.249 |
|  | Genotype | 7 | 31.79 | 481 | 131.72 | 16.52 | **< 2.2e^-16^** |
|  | Cup | 7 | 3.81 | 474 | 127.91 | 1.98 | 0.0567 |
|  | Treatment:genotype | 7 | 3.93 | 467 | 123.98 | 2.04 | **0.0486** |

**Table S11**. Pairwise post-hoc contrasts for linear models between sampled trait means of eight *Lemna minor* genotypes and treatment: unexposed populations (U) and populations exposed to *Spirodela polyrhiza* at low density (E) for 14 weeks. Contrasts were performed with the emmeans “contrast” function, and family-wise p-values were adjusted using the “mvt” method. SE = standard error, LCL = lower 95% confidence interval, UCL = upper 95% confidence level. P-values are bolded for significant relationships (p < 0.05). Sample sizes are as follows: root length: N = 447, 22-40 per treatment and genotype; raft size: N = 490, 24-40; frond area: N = 478, 21-40; root length/frond area: N = 470, 21-40.

| **Trait** | **Genotype** | **Plasticity**  **(Trait mean _E_ – Trait mean _U_)** | **SE** | **LCL** | **UCL** | **P-value** |
| --- | --- | --- | --- | --- | --- | --- |
| Root length (mm) | L-5500 | 2.29 | 1.55 | -0.77 | 5.36 | 0.141 |
|  | L-5576 | 5.48 | 1.46 | 2.59 | 8.37 | **0.0003** |
|  | L-7436 | 0.72 | 1.33 | -1.93 | 3.37 | 0.589 |
|  | L-8623 | 2.36 | 1.55 | -0.72 | 5.43 | 0.132 |
|  | L-8625 | -0.35 | 1.21 | -2.77 | 2.06 | 0.772 |
|  | L-8784 | -0.044 | 1.22 | -2.48 | 2.39 | 0.971 |
|  | L-9223 | -0.47 | 1.25 | -2.95 | 2.01 | 0.707 |
|  | L-9495 | -4.39 | 1.38 | -7.13 | -1.65 | **0.0020** |
|  |  |  |  |  |  |  |
| Raft size (# live fronds per raft) | L-5500 | 0.50 | 0.26 | -0.0026 | 1.00 | 0.0512 |
|  | L-5576 | 0.49 | 0.27 | -0.049 | 1.02 | 0.0751 |
|  | L-7436 | -0.19 | 0.23 | -0.63 | 0.26 | 0.407 |
|  | L-8623 | -0.47 | 0.26 | -0.98 | 0.053 | 0.0788 |
|  | L-8625 | -0.23 | 0.18 | -0.58 | 0.13 | 0.205 |
|  | L-8784 | -0.21 | 0.20 | -0.61 | 0.19 | 0.307 |
|  | L-9223 | -0.15 | 0.20 | -0.54 | 0.25 | 0.472 |
|  | L-9495 | 0.11 | 0.25 | -0.39 | 0.61 | 0.664 |
|  |  |  |  |  |  |  |
| Frond area (mm^2^) | L-5500 | 0.095 | 0.15 | -0.21 | 0.40 | 0.532 |
|  | L-5576 | 0.20 | 0.15 | -0.088 | 0.49 | 0.170 |
|  | L-7436 | 0.15 | 0.13 | -0.12 | 0.41 | 0.279 |
|  | L-8623 | -0.48 | 0.15 | -0.77 | -0.19 | **0.0016** |
|  | L-8625 | -0.13 | 0.12 | -0.38 | 0.12 | 0.287 |
|  | L-8784 | 0.25 | 0.12 | 0.0039 | 0.50 | **0.047** |
|  | L-9223 | 0.11 | 0.12 | -0.13 | 0.36 | 0.361 |
|  | L-9495 | -0.22 | 0.14 | -0.50 | 0.056 | 0.116 |
|  |  |  |  |  |  |  |
| Root length : frond area  (mm/mm^2^) | L-5500 | 0.70 | 0.81 | -0.89 | 2.30 | 0.385 |
|  | L-5576 | 1.27 | 0.75 | -0.23 | 2.76 | 0.0957 |
|  | L-7436 | 0.089 | 0.69 | -1.28 | 1.46 | 0.891 |
|  | L-8623 | 2.69 | 0.77 | 1.17 | 4.21 | **0.0007** |
|  | L-8625 | 0.32 | 0.64 | -0.95 | 1.60 | 0.615 |
|  | L-8784 | -0.91 | 0.64 | -2.17 | 0.35 | 0.155 |
|  | L-9223 | -0.57 | 0.65 | -1.86 | 0.72 | 0.383 |
|  | L-9495 | -1.69 | 0.72 | -3.12 | -0.26 | **0.0208** |

**Table S12.** ANOVA results of linear models comparing measured traits in *Lemna minor* among genotypes. Models in part (A) are derived from linear mixed models with an interaction between genotype and treatment, with cup replicate ID as a random factor. Treatments included isogenic + low-nutrient, isogenic + high-nutrient, polygenic + low-nutrient, polygenic + low-nutrient + exposure to the competitor *Spirodela polyrhiza* over 14 weeks (root length: N = 802, 20-40 per treatment and genotype; frond area: N = 798, 20-40; root length/frond area: N = 790, 20-40). The model from (B) was fitted with a quasi-Poisson family generalized linear model with an interaction between treatment and genotype and cup replicate ID as a fixed factor (N = 810, 20-40). (C) Was modelled as a simple linear regression with genotype predicting low-nutrient, low-density growth rate, calculated as the number of fronds produced after five days divided by the initial number of fronds across two rafts in small cups (N = 40, 5 per genotype). Bolded values indicate p < 0.05. Resid. = residual. Df = degrees of freedom. Num = numerator. Den = denominator.

| **A.** |  |  |  |  |  |  |  |
| --- | --- | --- | --- | --- | --- | --- | --- |
| Trait |  | Sum Sq. | Mean Sq. | NumDf | DenDf | F-Value | P-Value |
| Ln(  Root length (mm)) | Genotype | 164.82 | 23.55 | 7 | 765.74 | 74.29 | **< 2.2e^-16^** |
|  | Treatment | 104.70 | 34.90 | 3 | 11.30 | 110.11 | **1.2e^-8^** |
|  | Genotype:treatment | 46.05 | 2.19 | 21 | 762.65 | 6.92 | **< 2.2e^-16^** |
|  |  |  |  |  |  |  |  |
| Ln(  Frond area (cm^2^)) | Genotype | 12.46 | 1.78 | 7 | 762.17 | 39.19 | **< 2.2e^-16^** |
|  | Treatment | 50.57 | 16.86 | 3 | 10.65 | 371.23 | **4.6e^-11^** |
|  | Genotype:treatment | 13.34 | 0.64 | 21 | 761.04 | 13.99 | **< 2.2e^-16^** |
| Root length/ frond area (mm/  mm^2^) | Genotype | 3702.3 | 528.90 | 7 | 754.61 | 68.77 | **< 2.2e^-16^** |
|  | Treatment | 866.7 | 288.91 | 3 | 14.25 | 37.56 | **5.1e^-7^** |
|  | Genotype:treatment | 1529.5 | 72.83 | 21 | 752.57 | 9.47 | **< 2.2e^-16^** |
| **B.** |  |  |  |  |  |  |  |
| Raft size  (# live fronds per raft) |  | Df | Deviance | Resid. Df | Resid. Dev. | F-value | P-value |
|  | Genotype | 7 | 50.00 | 802 | 243.28 | 25.34 | **< 2.2e^-16^** |
|  | Treatment | 3 | 0.655 | 799 | 242.62 | 0.77 | 0.509 |
|  | Cup | 8 | 4.54 | 791 | 238.09 | 2.01 | **0.0425** |
|  | Genotype:treatment | 21 | 25.85 | 770 | 212.24 | 4.37 | **4.0e^-10^** |
| C. |  |  |  |  |  |  |  |
|  |  | Df | Sum Sq. | Mean Sq | - | F-value | P-value |
| Low-nutrient, low-density growth rate (λ_L_) | Genotype | 7 | 6.77 | 0.97 | - | 6.63 | **7.1e^-5^** |
|  |  |  |  |  |  |  |  |

**Table S13.** ANOVA results of linear models comparing measured traits between confirmed *Lemna minor* (L-5500, L-5576, L-8263, L-9495) and *Lemna x japonica* (L-7436, L-8625, L-8784) genotypes. Models in part (A) are derived from linear mixed models with an interaction between hybrid status and treatment, with cup replicate ID as a random factor. Treatments included isogenic + low-nutrient, isogenic + high-nutrient, polygenic + low-nutrient, polygenic + low-nutrient + exposure to the competitor *Spirodela polyrhiza* over 14 weeks (Frond area: N = 686, 60-119 per treatment and hybrid status; Root length/frond area: N = 682, 60-118). The model from (B) was fitted with a quasi-Poisson family generalized linear model (N = 698, 60-120). The models under section (C) are simple linear regressions (Root length: N = 60-119, **λ_L_:** N = 40, 5 per genotype). Bolded values indicate p < 0.05. Resid. = residual. Df = degrees of freedom. Num = numerator. Den = denominator.

| **A.** |  |  |  |  |  |  |  |
| --- | --- | --- | --- | --- | --- | --- | --- |
| Trait |  | Sum Sq. | Mean Sq. | NumDf | DenDf | F-Value | P-Value |
| Ln (  Frond area (mm^2^)) | Hybrid status | 5.03 | 5.03 | 1 | 673.05 | 79.00 | **< 2.2e^-16^** |
|  | Treatment | 52.24 | 17.41 | 3 | 10.46 | 273.35 | **3.2e^-10^** |
|  | Hybrid status:treatment | 4.88 | 1.63 | 3 | 673.09 | 25.54 | **1.2e^-15^** |
| Root length/ frond area (mm/  mm^2^) | Hybrid status | 426.78 | 426.78 | 1 | 671.67 | 38.23 | **1.1e^-9^** |
|  | Treatment | 1051.76 | 350.59 | 3 | 14.30 | 31.41 | **1.5e^-6^** |
|  | Hybrid status:treatment | 227.02 | 75.67 | 3 | 671.14 | 6.78 | **0.00017** |
|  |  |  |  |  |  |  |  |
| **B.** |  |  |  |  |  |  |  |
| Raft size  (# live fronds per raft) |  | Df | Deviance | Resid. Df | Resid. Dev. | F-value | P-value |
|  | Hybrid status | 1 | 25.88 | 696 | 230.06 | 75.19 | **<2.2e^-16^** |
|  | Treatment | 3 | 0.45 | 693 | 229.61 | 0.43 | 0.728 |
|  | Hybrid status:treatment | 3 | 2.88 | 690 | 226.73 | 2.79 | **0.0396** |
| **C.** |  |  |  |  |  |  |  |
|  |  | Df | Sum Sq. | Mean Sq | - | F-value | P-value |
| Ln(Root length (mm)) | Hybrid status | 1 | 1.45 | 1.45 |  | 4.43 | **0.036** |
|  | Treatment | 3 | 273.07 | 91.02 |  | 278.38 | **<2.2e^-16^** |
|  | Hybrid status:treatment | 3 | 6.16 | 2.05 |  | 6.28 | **0.00033** |
|  |  |  |  |  |  |  |  |
| Low-nutrient, low-density growth rate (λ_L_) | Hybrid status | 1 | 0.038 | 0.038 |  | 0.17 | 0.69 |
|  |  |  |  |  |  |  |  |

**Table S14**. Summary of linear regression results from univariate models between exposure-driven selection or intraspecific selection and trait means and plasticities measured in *Lemna minor* genotypes (N = 8). Range shifter exposure-driven selection is the difference in estimated marginal means for ΔRA (changes in relative abundance for *L. minor* resident genotypes over 14 weeks) between resident populations exposed to (E) and unexposed to (U) *Spirodela polyrhiza* at low density (Exposure selection % = ΔRA _E_ - ΔRA _U_). Intraspecific selection is the change in estimated marginal means of relative abundance of *L. minor* genotypes over 14 weeks in populations unexposed to *S. polyrhiza*. λ = per capita low-density, low-nutrient growth rate; RL = root length (mm); FA = frond area (mm^2^); RS = raft size (count); RLFA = root length to frond area ratio (mm/mm^2^).

| Response | Predictors | Estimate | SE | p | R^2^-adjusted |
| --- | --- | --- | --- | --- | --- |
| Exposure selection | Intercept | -4.49 | 1.390 | 0.018 | 0.59 |
|  | **λ_L_** | **2.371** | **0.718** | **0.016** |  |
|  | Intercept | -1.355 | 1.201 | 0.302 | 0.06 |
|  | Low-nutrient RL | 0.047 | 0.039 | 0.270 |  |
|  | Intercept | -1.425 | 1.247 | 0.297 | 0.07 |
|  | High-nutrient RL | 0.032 | 0.026 | 0.267 |  |
|  | Intercept | -1.362 | 2.127 | 0.546 | -0.088 |
|  | Low-nutrient FA | 0.388 | 0.590 | 0.535 |  |
|  | Intercept | 0.203 | 1.930 | 0.920 | -0.164 |
|  | High-nutrient FA | -0.030 | 0.275 | 0.917 |  |
|  | Intercept | 0.707 | 2.702 | 0.802 | -0.15 |
|  | Low-nutrient RS | -0.252 | 0.947 | 0.799 |  |
|  | Intercept | 1.061 | 2.195 | 0.646 | -0.12 |
|  | High-nutrient RS | -0.364 | 0.733 | 0.638 |  |
|  | Intercept | -0.409 | 1.159 | 0.736 | -0.14 |
|  | Low-nutrient RLFA | 0.0457 | 0.117 | 0.711 |  |
|  | Intercept | -2.291 | 1.214 | 0.108 | 0.30 |
|  | High-nutrient RLFA | 0.340 | 0.171 | 0.0937 |  |
|  | Intercept | -2.350 | 2.887 | 0.447 | -0.05 |
|  | RL plasticity | 0.030 | 0.036 | 0.441 |  |
|  | Intercept | 0.172 | 1.881 | 0.930 | -0.16 |
|  | FA plasticity | -0.00312 | 0.0330 | 0.928 |  |
|  | Intercept | 3.487 | 6.149 | 0.591 | -0.11 |
|  | RS plasticity | -0.105 | 0.185 | 0.590 |  |
|  | Intercept | 2.487 | 2.030 | 0.267 | 0.076 |
|  | RLFA plasticity | -0.0434 | 0.0346 | 0.256 |  |
|  |  |  |  |  |  |
| Intraspecific selection | Intercept | -3.465 | 5.921 | 0.580 | -0.10 |
|  | λ_L_ | 1.831 | 3.057 | 0.571 |  |
|  | Intercept | -3.473 | 3.152 | 0.313 | 0.05 |
|  | Low-nutrient RL | 0.120 | 0.101 | 0.281 |  |
|  | Intercept | -6.778 | 2.120 | 0.019 | 0.60 |
|  | **High-nutrient RL** | **0.151** | **0.044** | **0.014** |  |
|  | Intercept | -2.539 | 5.653 | 0.669 | -0.13 |
|  | Low-nutrient FA | -0.723 | 1.569 | 0.661 |  |
|  | Intercept | -10.727 | 2.221 | 0.00291 | 0.77 |
|  | **High-nutrient FA** | **1.584** | **0.317** | **0.00246** |  |
|  | Intercept | 10.147 | 5.714 | 0.126 | 0.24 |
|  | Low-nutrient RS | -3.616 | 2.002 | 0.121 |  |
|  | Intercept | 8.576 | 4.619 | 0.113 | 0.27 |
|  | High-nutrient RS | -2.938 | 1.543 | 0.106 |  |
|  | Intercept | -1.915 | 2.942 | 0.539 | -0.074 |
|  | Low-nutrient RLFA | 0.214 | 0.298 | 0.500 |  |
|  | Intercept | -2.780 | 3.907 | 0.503 | -0.067 |
|  | High-nutrient RLFA | 0.412 | 0.549 | 0.481 |  |
|  | Intercept | -10.002 | 6.798 | 0.192 | 0.15 |
|  | RL plasticity | 0.128 | 0.086 | 0.187 |  |
|  | Intercept | -10.366 | 2.220 | 0.00334 | 0.76 |
|  | **FA plasticity** | **0.189** | **0.0390** | **0.00288** |  |
|  | Intercept | 5.044 | 16.361 | 0.768 | -0.15 |
|  | RS plasticity | -0.152 | 0.493 | 0.768 |  |
|  | Intercept | 7.395 | 5.093 | 0.197 | 0.15 |
|  | RLFA plasticity | -0.129 | 0.0868 | 0.187 |  |
|  |  |  |  |  |  |

**Table S15**. ANOVA summaries of linear regression models of finite re-exposure growth rate (λ_RE_) of *Spirodela polyrhiza*, fronds, floating (juvenile) turions, and submerged (mature) turions, 15 and 21 days after being introduced to previously unexposed/ naïve (n = 18) and *Lemna minor* populations previously exposed to *S. polyrhiza* at low density (n =17). λ_RE_ was calculated as the final divided by the initial abundance of each tissue type. Mold presence was quantified as visible mold presence (binary) in week 3 or weeks 1 and 3 of the experiment. In models labelled “M1”, mold extent (0-10) was used as a random covariate, while models labelled “M2” used *Lemna minor* population size in week 14 was used as a random variable. For floating and submerged turions, the initial abundance was set to 1 for mathematical feasibility. “DF” = degrees of freedom. Df = degrees of freedom. Num = numerator. Den = denominator. Model (A) is a simple linear regression, while models in (B) are linear mixed models.

| **A.** |  | **DF** | **Sum Sq.** | **Mean Sq.** | **-** | **F-value** | **P-value** |
| --- | --- | --- | --- | --- | --- | --- | --- |
| **Fronds M1** | Treatment | 1 | 0.0421 | 0.042 | - | 1.434 | 0.2355 |
|  | Time | 1 | 0.0077 | 0.008 | - | 0.264 | 0.6093 |
|  | Mold presence (week 1-3) | 1 | 0.0012 | 0.001 | - | 0.037 | 0.8490 |
|  | Treatment x Time | 1 | 0.0091 | 0.009 | - | 0.310 | 0.5800 |
|  | Residuals | 65 | 1.907 | 0.0029 | - | - | - |
|  |  |  |  |  |  |  |  |
| B. |  | **Sum Sq** | **Mean Sq** | **Num DF** | **Den**  **DF** |  |  |
| **Fronds M2** | Treatment | 0.0050 | 0.005 | 1 | 30.43 | 0.273 | 0.6051 |
|  | Time | 0.0067 | 0.007 | 1 | 32.08 | 0.363 | 0.5510 |
|  | Mold presence (week 3) | 0.2908 | 0.291 | 1 | 35.75 | 1.583 | 0.2164 |
|  | Treatment x time | 0.0084 | 0.008 | 1 | 32.08 | 0.458 | 0.5035 |
|  |  |  |  |  |  |  |  |
| **Floating**  **turions M1** | Treatment | 0.0188 | 0.019 | 1 | 64.06 | 0.318 | 0.5750 |
|  | Time | 0.1472 | 0.147 | 1 | 61.68 | 2.488 | 0.1198 |
|  | Mold presence (week 3) | 0.0091 | 0.009 | 1 | 64.03 | 0.153 | 0.6967 |
|  | Treatment x Time | 0.0015 | 0.002 | 1 | 61.68 | 0.026 | 0.8729 |
|  |  |  |  |  |  |  |  |
| **Floating turions M2** | Treatment | 0.0076 | 0.008 | 1 | 32.02 | 0.287 | 0.5961 |
|  | Time | 0.1337 | 0.134 | 1 | 33.05 | 5.069 | **0.0311** |
|  | Mold presence (week 3) | 0.0184 | 0.018 | 1 | 39.16 | 0.698 | 0.4085 |
|  | Treatment x Time | 0.0026 | 0.003 | 1 | 33.05 | 0.010 | 0.7541 |
|  |  |  |  |  |  |  |  |
| **Submerged turions M1** | Treatment | 10.565 | 10.565 | 1 | 64.56 | 5.292 | **0.02466** |
|  | Time | 39.257 | 39.257 | 1 | 63.43 | 19.665 | **3.743e^-5^** |
|  | Mold presence (week 3) | 0.264 | 0.264 | 1 | 64.54 | 0.132 | 0.7176 |
|  | Treatment x Time | 5.429 | 5.429 | 1 | 63.43 | 2.719 | 0.1041 |
| **Submerged turions M2** | Treatment | 5.553 | 5.55 | 1 | 30.02 | 5.370 | **0.0275** |
|  | Time | 33.882 | 33.88 | 1 | 31.21 | 32.769 | **2.631e^-6^** |
|  | Mold presence (week 3) | 0.010 | 0.01 | 1 | 36.68 | 0.001 | 0.9216 |
|  | Treatment x Time | 3.765 | 3.77 | 1 | 31.21 | 3.641 | 0.0656 |
|  |  |  |  |  |  |  |  |
| **All life stages M1** | Treatment | 0.0102 | 0.010 | 1 | 64.10 | 0.147 | 0.7031 |
|  | Time | 0.0014 | 0.001 | 1 | 61.71 | 0.020 | 0.8888 |
|  | Mold presence (week 3) | 0.0110 | 0.011 | 1 | 64.10 | 0.159 | 0.6918 |
|  | Treatment x Time | 0.0402 | 0.040 | 1 | 61.71 | 0.579 | 0.4498 |
| **All life stages M2** | Treatment | 0.0058 | 0.006 | 1 | 30.34 | 0.194 | 0.6631 |
|  | Time | 0.0008 | 0.001 | 1 | 31.24 | 0.027 | 0.8716 |
|  | Mold presence (week 3) | 0.0142 | 0.014 | 1 | 37.92 | 0.471 | 0.4968 |
|  | Treatment x Time | 0.0359 | 0.036 | 1 | 31.24 | 1.195 | 0.2827 |

**Table S16.** Summary of estimated marginal means of linear regression models of finite re-exposure growth rate λ_RE_ of *Spirodela polyrhiza*, fronds, floating (juvenile) turions, and submerged (mature) turions, 15 and 21 days after being introduced to previously unexposed/ naïve (U, n = 18) and *Lemna minor* populations previously exposed to *S. polyrhiza* at low density (E, n =17). λ_RE_ was calculated as the final divided by the initial abundance of each tissue type. Each model treated mold presence in week 3 (binary) and *Lemna minor* population size in week 14 as fixed and random effects, respectively. For floating and submerged turions, the initial abundance was set to 1 for mathematical feasibility. “CI” = 95 % confidence interval. “EMM” = estimated marginal mean (%). “DF” = degrees of freedom.

| Life stage | Week | Treatment | EMM (%) | DF | SE  (%) | Lower CI (%) | Upper CI  (%) |
| --- | --- | --- | --- | --- | --- | --- | --- |
| Fronds | 2 | U | 0.850 | 43.5 | 0.0511 | 0.747 | 0.953 |
|  |  | E | 0.802 | 57.6 | 0.0427 | 0.716 | 0.887 |
|  | 3 | U | 0.848 | 43.5 | 0.0511 | 0.745 | 0.951 |
|  |  | E | 0.844 | 57.6 | 0.0427 | 0.758 | 0.929 |
|  |  |  |  |  |  |  |  |
| Submerged turions | 2 | U | 0.855 | 40.1 | 0.434 | -0.023 | 1.73 |
|  |  | E | 1.39 | 55.6 | 0.354 | 0.681 | 2.10 |
|  | 3 | U | 1.80 | 40.1 | 0.434 | 0.989 | 2.67 |
|  |  | E | 3.27 | 55.6 | 0.354 | 2.56 | 3.98 |
|  |  |  |  |  |  |  |  |
| Floating turions | 2 | U | 1.23 | 39.0 | 0.0728 | 1.09 | 1.38 |
|  |  | E | 1.18 | 54.9 | 0.0587 | 1.065 | 1.30 |
|  | 3 | U | 1.13 | 39.0 | 0.0728 | 0.986 | 1.28 |
|  |  | E | 1.11 | 54.9 | 0.0587 | 0.989 | 1.22 |
|  |  |  |  |  |  |  |  |
|  |  |  |  |  |  |  |  |
| All life stages | 2 | U | 1.29 | 38.3 | 0.0806 | 1.13 | 1.46 |
|  |  | E | 1.28 | 54.5 | 0.0644 | 1.15 | 1.41 |
|  | 3 | U | 1.25 | 38.3 | 0.0806 | 1.09 | 1.42 |
|  |  | E | 1.34 | 54.5 | 0.0644 | 1.21 | 1.46 |
