# Supplementary material for: Simulated Range Expansion Suggests Rapid Change in Biotic Resistance to a Range‐Shifting Competitor
Source: Ecol Evol. 2026 Jul 29;16(8):e74096. doi: 10.1002/ece3.74096 (PMC13420321; doi:10.1002/ece3.74096)
Supplement: Supplementary file 2 — Data S1: Supplementary Methods: Frond area estimation protocol. [file ECE3-16-e74096-s001.docx]

**Simulated range expansion suggests rapid evolution of biotic resistance to a range-shifting competitor**

**Supplementary Methods: Frond area estimation protocol**

1. **Imaging**

We taped a metal stand (9 in tall) with a base (6 in length) and a Sigma-Aldrich 3-prong clamp to a countertop in an indoor location with bright, even, and consistent lighting (away from windows). We placed a lightbox (not turned on) beneath the clamp configuration for a white background (a thick white sheet of paper would also suffice). We filled a 1-oz plastic portion cup with 23 mL of deionized water, placed a single duckweed raft in the cup, and placed it on the lightbox under the clamp. We affixed an iPhone 13 Pro to the 3-pronged clamp and adjusted the clamp height to select an ideal field of view. This field of view contained only the 1-oz cup with duckweed and the lightbox, excluding any other coloured objects that could interfere with colour selection during later image-processing steps. The height that we selected forced the iPhone to use the macro photography mode. We then marked the clamp's vertical position with tape and the centre of the field of view on the lightbox. Each time the iPhone was affixed to the clamp, we used the level tool in the “Measure” app to ensure the phone was consistently level (0 degrees).

We imaged each sampled duckweed raft in 1-oz portion cups placed at the approximate centre of the field of view. Within each image, we also placed a ruler at water level for a reference scale. By imaging the genotypes (n = 8) and replicates (n = 8) in a consistent order, we used a simple R script to rename the JPEG image files with informative filenames that detail the genotype, treatment, replicate, and imaging date.

1. **Estimating frond area**

We initially estimated frond area by using a batch-processing protocol to measure raft area (Usui, 2023) and dividing this by the raft size (e.g. Hess et al., 2022). However, we found that this method tended to overestimate frond area for some genotypes more than others. We therefore devised a protocol to manually estimate frond area in ImageJ. For each sample, we selected two mature fronds and estimated their areas by tracing outlines with the polygon selection tool. These data were collected only by the lead author to avoid measurement inconsistencies. We reset the spatial scale before measuring new samples from each cup replicate, as the imaging setup tended to differ slightly between replicates despite efforts to maintain scale consistency.

To select two fronds per raft to measure, we used a combination of random and targeted sampling. For instance, if two fronds in the sample raft lacked nail polish dots, we assumed they represented the newest generation and selected them for measurement to serve as the endpoint conditions of the experiment. We also removed some fronds from consideration for sampling. First, we excluded juvenile fronds (lacking a tapering base) to reduce the potential for frond maturity to bias estimates. Additionally, we did not consider fronds that were unhealthy (white or yellow). Finally, we used the sample() function in R to select from the remaining fronds that matched the selection criteria, numbering the selected fronds from those closest to the bottom of the image.

In measuring fronds from isogenic, low-nutrient and high-nutrient treatments, we intentionally biased selections towards smaller and larger fronds, respectively. For the former case, smaller fronds are likely the newest in birth order, as frond area decreases on low-nutrient media, and we wanted to avoid measuring individuals developed under high-nutrient conditions. Similarly, for the isogenic, high-nutrient populations, we tended to select larger fronds to avoid maturity effects. Therefore, these data should not be compared across treatments but only between genotypes, as intended. A detailed protocol is outlined below.

**Requirements:**

- ImageJ (we used version 1.53t, but the following procedure is likely compatible with newer and older versions)
- JPEG files of imaged duckweed rafts with informative file names
- Empty spreadsheet with three columns: “placeholder_value”, “filename”, “area_[insert unit]_2”

**Procedure:**

1. Open ImageJ
2. *Upon the first time opening the application:* In the main menu, select “**Analyze” > “Set Measurements” > tick: area, display label.**
3. Setting a global scale:
   1. Open one of the images **(“File” > “Open…” > select the first photo in the series > “Open”)**
   2. Select the straight line tool from the toolbar
   3. Draw a line between two known increment measurements on the ruler in the image, as directly and straight as possible between the two points. *Tip: Use the “+” key with the cursor over the ruler in the image to zoom in for better accuracy.*
   4. In the main menu, select **“Analyze” > “Set Scale…” > “Known distance” =** the real-world increment you measured on the ruler using the straight line tool (e.g. 1), unit = the unit you measured (e.g. cm) **> TICK the box that says GLOBAL > “Ok”**
   5. The scale is now set for the duration the ImageJ application is open. Assuming that images were taken consistently, this scale should be reasonable for many images. If images were taken on different days, or if the camera was moved between image series, consider repeating this step and setting a scale for each batch of images on each day to avoid scale inconsistencies caused by slight movements in camera position.
4. With the image still open, use the **“+”** key to zoom in on fronds.
5. Select two fronds to measure.
   1. Count the number of fronds that…
      1. Are mature (even if attached to a parent, they show a taper at the base or are similar size to other fronds present).
      2. Are healthy (contain mostly green tissue).
   2. If two fronds are present for which visual cues indicate are the latest in the birth order, select these (no random selection required).
   3. If one frond is present for which visual cues indicate is the latest in the birth order, select this as the first frond, and randomly select the second.
   4. For the remaining samples, use the sample() function in R.
      1. Input the number of fronds to sample from (e.g. 4), and the sample size (e.g. 2): sample(1:4, 2).
      2. To map this selection to fronds in the image, number the fronds beginning with those located closest to the bottom of the image.
6. Select the **“Polygon selections”** tool from the toolbar.
7. Use this tool to outline the frond with reasonable precision.
   1. Click on an area along the frond outline to begin.
   2. Place points around the frond perimeter each time by clicking the image.
      1. The number of points required may vary depending on the frond's curvature and size, as well as the desired precision. We used ~ 10-20 points per frond.
      2. For fronds attached to a daughter or parent, use colour variation to determine where this intersection occurs. We did not include the tips of daughter fronds budding from the meristematic pockets in the selection of the parental frond area (see example below).
      3. If a tracing error is made, close the selection by clicking on the beginning point and then clicking somewhere else on the image.
      4. To finish the outline, click on the initial point.


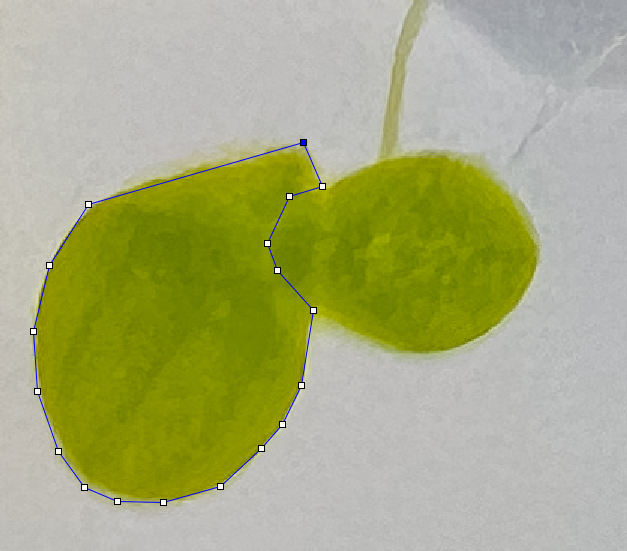

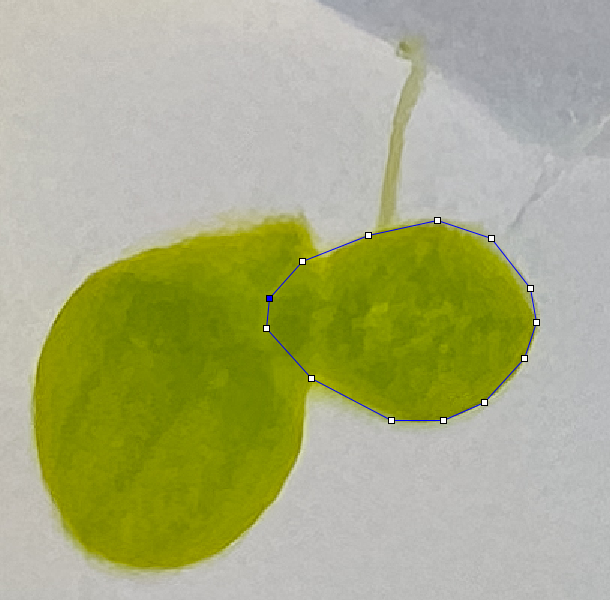


1. Measure the selected area: **“Analyze” → “Measure” (or Command “M”)**.
   1. The measured area should appear as the square of the units you set the scale with.
   2. Copy these measurements to the spreadsheet.
   3. If the measurement is zero or is an extremely small number, ensure that the polygon selection was closed.
2. **Repeat 6-7 for the second frond** if one matching the criteria exists for the sampled raft.
3. Re-set the scale between image series (e.g. between samples from different replicates) to avoid scale inconsistencies.
4. Once all measurements are completed, use R to…
   1. Separate the label or filename column from the data collection spreadsheet into informative descriptors of each data point (e.g. genotype, replicate, sample number, date).
   2. Average across the two measurements for each image to obtain one frond area estimate per sampled raft.
